# Understanding and translating evolutionary adaptations in animals exposed to contaminated diets to guide strategies against human foodborne disease: a prioritisation framework

**DOI:** 10.3389/fimmu.2026.1883529

**Published:** 2026-10-19

**Authors:** Muhammad Abaidullah, Yuan Ji, Qinmiao Huang, Marco Scarpa, Jing Li, Xiaojun Xiao, Damo Xu

**Affiliations:** 1 Department of Respiratory Medicine and Allergy, The Third Affiliated Hospital (The Affiliated Luohu Hospital) of Shenzhen University, State Key Laboratory of Respiratory Disease Shenzhen University Division, Shenzhen University Medical School, Shenzhen University, Shenzhen, Guangdong, China; 2 Institute of Allergy and Immunology, Shenzhen Key Laboratory of Allergy and Immunology, Guangdong Engineering Technology Research Center for Allergen Standardization, Shenzhen University, Shenzhen, Guangdong, China; 3 Department of Allergy and Clinical Immunology, State Key Laboratory of Respiratory Disease, The First Affiliated Hospital of Guangzhou Medical University, Guangzhou, China; 4 Shenzhen Institute for Drug Control (Shenzhen Testing Center of Medical Devices), Nanshan, Shenzhen, China; 5 Department of Surgical, Oncological and Gastroenterological Sciences (DISCOG), University of Padova, Padova, Italy

**Keywords:** animal adaptations, biomimetic detoxification, colonisation resistance, disease tolerance, foodborne diseases, host-microbiome coevolution, pathogen biocontrol, trained immunity

## Abstract

**Background:**

Humans face increasing vulnerability to foodborne and infectious diseases despite medical advances, while certain animal lineages cope with pathogen-laden diets and environments under the particular ecological pressures that selected their defences. Animals are not universally resistant, and wildlife and livestock outbreaks of Salmonella, Campylobacter, Vibrio and mycotoxins are documented; but certain lineages have evolved tolerance or rapid-clearance mechanisms with minimal pathology. This disparity arises from evolutionary adaptations in animals, contrasting with human reliance on hygiene and antibiotics, which contribute to antimicrobial resistance and zoonotic spillovers, while modern sanitation has also delivered dramatic reductions in waterborne disease mortality.

**Aim of review:**

This review aims to synthesize evidence on animal disease resistance mechanisms and translate these insights into clinical strategies for improving human health, addressing gaps in comparative biology and combating emerging threats like antimicrobial resistance. We introduce the Biomimetic Immunity Continuum, a translational framework for comparative immunology built on an explicit selection rule that rejects candidate adaptations as well as ranking them; four are rejected here. It maps animal defence pillars (genetic, ecological, behavioural, physiological, microbiome) along a spectrum from prevention to molecular tuning to therapeutic replacement, prioritizing feasibility, reversibility, safety, and scalability. These criteria are applied as three exclusion gates and four ranked criteria, with candidates ordered on the limiting step rather than on a composite numerical score. We predict that, among adaptations acting on conserved or reachable human pathways, translational potential depends more on conservation, accessibility, safety and deliverability than on the size of the phenotype in the source animal. Throughout, we distinguish avoidance (reduction of exposure), resistance (reduction of pathogen or toxin burden), tolerance (reduction of health cost per unit burden) and resilience (the composite property emerging when these operate together), because these axes carry different translational and safety implications.

**Key scientific concepts of review:**

Animals employ multifaceted defences: genetic (e.g., immune gene evolution), ecological (e.g., habitat avoidance), behavioural (e.g., grooming), physiological (e.g., vultures’ hyperacidic stomachs neutralizing anthrax), immunological (e.g., epigenetic reprogramming), and microbial (e.g., microbiome-mediated exclusion). These synergize to prevent infections, as seen in bats tolerating high viral loads with minimal immunopathology. Candidate human applications differ widely in maturity: trained immunity via BCG and defined live biotherapeutic products are already in human use, dietary interventions mimicking animal microbiomes have reached controlled trials, and heat-shock induction has human data but no controlled trial against a foodborne endpoint. We separate mechanistic inspiration from candidate intervention throughout, and the evidence level attached to each proposal says which it is. Bridging evolutionary and clinical science offers candidate approaches to disease prevention. Every proposal is classified by translational maturity on a five-level scale from approved human use to source-species observation alone; this records maturity rather than certainty or demonstrated efficacy. Animal exemplars are included only where they meet stated relevance criteria for foodborne hazards as defined by the World Health Organization, which treats infectious and chemical hazards together.

## 1 Introduction

Foodborne illnesses remain one of the most persistent threats to human health, with ~600 million cases of disease and 420,000 deaths worldwide each year (1), despite the advances in sanitation, food safety, and antimicrobial therapy. This stubborn vulnerability contrasts with the defensive capacity observed in some animal lineages (2–4). For example, wild animals routinely ingest food and water heavily contaminated with enteric pathogens, yet suffer almost no clinical consequences (5–8). It is important to note that animals are not universally resistant; documented wildlife and livestock outbreaks of Salmonella, Campylobacter, Vibrio, and mycotoxins demonstrate real vulnerability in many species (9–12). However, certain lineages have evolved remarkable tolerance or rapid-clearance mechanisms with minimal pathology in their specific ecological niches. It is these context-specific adaptations that offer selective biomimetic lessons rather than any claim of blanket superiority.

Environmental factors have profoundly shaped human evolution, though mechanisms remain incompletely understood (13). The Neolithic Revolution ~10,000–12,000 years ago marked the transition from hunter-gatherer to agricultural lifestyle (14). Yet the most dramatic transformation occurred over the past ~200 years with industrialization and urbanization, creating an environment profoundly mismatched with our evolutionary adaptations (15, 16). These changes have transformed human genetics, immunity, and metabolism with both positive and negative consequences (17).

Positively, they enabled conquest of ancestral scourges: famine, rampant infection and trauma, thereby relaxing raw “survival” as the dominant evolutionary pressure and unleashing rapid population growth, longevity, and cognitive specialization (18). Negatively, the speed of environmental change far outpaced genetic evolution, producing widespread mismatch diseases (16, 19). Many such disorders, metabolic, autoimmune, allergic, and infectious, have now been identified. For example, enhanced food availability combined with decreased physical activity has driven dramatic increases in obesity, type 2 diabetes, and cardiovascular disease (19, 20).

The immune system requires continuous, diverse microbial exposure, including harmless commensals and parasitic “old friends”, for proper calibration (21, 22). However, the hygiene hypothesis (now “old friends” framework) explains how modern hygiene, urbanization, and antibiotic overuse have depleted these exposures, yielding an under-trained, dysregulated immune system (22, 23). While modern sanitation and hygiene have delivered dramatic, life-saving reductions in waterborne and foodborne mortality (cholera, typhoid, dysentery, etc.) (24), the ‘old friends’ framework highlights important complementary trade-offs: depletion of diverse microbial exposures contributes to immune dysregulation underlying allergies, autoimmunity, and inflammaging. The review advocates targeted hygiene approaches that control genuine pathogens while preserving beneficial exposures where safe.

While this man-made environment reduced some infectious diseases, it also relaxed pathogen-driven selective pressures that once maintained multilayered defences, leaving modern humans reliant on cultural and technological defences where some animal lineages retain layered biological ones; Section 8.4 sets out why this is not a general claim about defensive capacity (25). Animals, under relentless pathogen pressure, evolved synergistic defence networks spanning genetics, physiology, immunity, and behaviour (3, 26–32).

Here we synthesise multidisciplinary literature into a framework built on five pillars of animal defence against ingested hazards: evolutionary genetic innovations, ecological drivers, behavioural and dietary strategies, extreme digestive chemistry with specialised immune mechanisms, and gut microbiome–host coevolution. We propose the Biomimetic Immunity Continuum, which ranks candidate interventions from those acting before ingestion or in the gut lumen, such as dietary phytochemicals and defined microbial consortia, to those requiring action on a conserved human pathway, such as inflammasome-directed agents and engineered antimicrobial peptides. Among adaptations acting on pathways humans still carry or can reach, the ranking follows conservation, accessibility, safety and deliverability rather than the magnitude of the animal phenotype. The Continuum is a decision procedure rather than a mechanistic model; Section 1.5 sets out how it differs from evolutionary medicine, ecological immunology, trained immunity and biomimetics, and Section 10 examines the selective consequences of acting on it.

The review is in three parts. Section 1 defines the terms, states what an animal example must satisfy to be included, sets out the six questions asked of every adaptation, and describes the two scales on which evidence is graded. Section 2 summarises the foodborne hazards themselves, and Sections 3 to 7 present the five pillars, each proposal carrying its evidence grade. Sections 8 to 12 test the framework rather than illustrate it: they work through the adaptations our own criteria reject, what each adaptation costs the animal carrying it, which solutions have evolved more than once, what selection our proposals would impose on pathogens, the limits of captive and laboratory evidence, and the scope and limits of trained immunity.

## 2 Framework, scope and evidence standards

### 2.1 Avoidance, resistance, tolerance and resilience: a conceptual scaffold

Animals defend themselves against contaminated food in three different ways, and we keep them separate throughout this review because they lead to different treatments. Avoidance means not eating the contaminated food at all: animals detect and reject it, or feed where contamination is rare (33). Resistance means clearing the pathogen after it has been swallowed, so fewer survive in the gut. Tolerance means staying well even though the pathogen is still present; the animal carries the same number of bacteria but suffers less damage from them. Tolerance is measured by plotting how sick an animal becomes against how many pathogens it carries, and a tolerant animal stays healthier at every level (34–38). Two animals can show the same survival for opposite reasons: one cleared the infection, the other endured it.

Resilience is our word for the combination. A resilient animal keeps functioning normally, or recovers quickly, after eating something harmful, whether because it avoided the hazard, cleared it, withstood it, or repaired the damage afterwards. It is not a fourth mechanism but what happens when several work together. Resilience in this sense is always relative to a particular hazard in a particular environment. We therefore use it to describe a measured outcome in a named context, and not as a label for animals as a class. No lineage is resilient in general, and an animal that withstands one ingested hazard may be highly vulnerable to another it did not evolve with, as Section 8.4 sets out. The animals examined here are those that combine more than one of these strategies against the specific hazards that selected them.

The distinction matters for treatment. Drugs that copy resistance kill pathogens, so bacteria evolve around them, which is the same antimicrobial resistance problem these approaches are meant to solve. Drugs that copy tolerance put no such pressure on the pathogen, but a patient may stay infectious for longer, which moves risk from the individual to the people around them (39). Avoidance acts before anyone is exposed and is the safest of the three. A review that mixes these three together cannot say which is preferable.

One limitation should be stated. These definitions were developed for living pathogens that multiply inside the host, whereas much foodborne illness comes from chemicals that do not multiply, such as mould toxins. For these we treat enzymes that destroy the toxin, and clays that bind it, as the equivalent of resistance, because they reduce how much toxin is present. We treat a stronger gut barrier and better antioxidant defence as the equivalent of tolerance, because they reduce the damage each unit of toxin causes. Where a mechanism does both, as gut bacteria often do, we say so rather than force a choice.

### 2.2 Scope and inclusion criteria

This review uses the World Health Organization definition of foodborne disease, which counts both living agents and chemicals: bacteria, viruses and parasites carried in food, mould toxins, natural plant poisons, and chemical contaminants (40–43). This is wider than the bacteria-focused view common in food safety practice, and it decides which animals are relevant here. Cyanide-releasing compounds in plants and mould toxins are foodborne hazards under this definition, so animals that neutralize them are directly on topic rather than a side example.

We include an animal example for one of three reasons. Tier A: the animal eats the hazard, and that hazard is one humans also meet in food. Tier B: the animal faces a different problem but solves it in the gut, or through a pathway humans also use against foodborne hazards; these examples support the mechanism only and are not evidence about foodborne disease. Tier C: the example is not about food at all, but shows that a type of solution can be built. Every example is listed in **Table 1** with its tier, where the evidence came from, and how strong that evidence is, and examples meeting none of the three are excluded. Tier B and Tier C examples are marked in the text at first mention so their role is clear; an example carrying no tier label is Tier A. These tiers are the eligibility criteria for this review rather than a published classification system. We state them in full so that a reader can disagree with any individual placement, following guidance that a review drawing on very different kinds of study should state its inclusion rules in full (44, 45). Representative examples that met none of the three tiers, or that met a tier but were later removed by a gate in Section 1.4, are listed in **Supplementary Table S1** with the reason in each case. The tier therefore does two things at once. It decides whether an example enters the review at all, and it sets a limit on what that example may be used to claim. Everything that follows applies only to examples admitted here, and the next step grades the claims made about them.

**Table 1:** Inclusion tier, study setting and evidence level for every animal exemplar discussed in this review.

Exemplar	Tier	Basis for inclusion	Study setting	Evidence level	Gate outcome	Priority group	Notes
Vultures and wandering albatross (gastric hyperacidity)	A	Ingested carrion; enteric bacterial hazard	Wild, observational	E5	Fails G2	Excluded	Inference inverted in Section 8.2: a barrier to preserve, not to augment
Formicine ants (formic acid crop acidification)	A	Ingested food; selective microbial control	Laboratory	E5	Not ranked	—	Convergent with avian acidification (Section 9)
Caenorhabditis elegans (food avoidance)	A	Pre-ingestion avoidance of pathogenic bacteria	Laboratory	E4	Pass	Near-term	Avoidance axis, Section 1.1
Rodent Fpr3 (virulence peptide detection)	A	Pre-ingestion avoidance	Laboratory	E4	Pass	Near-term	Basis for VOC and biosensor translation
Murine T2R (quorum-sensing detection)	A	Pre-ingestion avoidance	Laboratory	E4	Pass	Near-term	Basis for biosensor translation
Canine VOC discrimination	A	Detection of contaminated food	Applied and field	E4	Pass	Near-term	Electronic-nose platforms in development; Limulus Factor C is the E1 precedent
Chimpanzee Vernonia amygdalina self-medication	A	Gastrointestinal parasites	Wild, observational	E2	Pass	Near-term	Plant secondary metabolites in trials
Ovine Sericea lespedeza	A	Gastrointestinal parasites	Field	E2	Pass	Near-term	Ethnoveterinary evidence base
Giant panda rhodanese (cyanogenic glycosides)	A	Cyanogenic glycosides are a recognised foodborne chemical hazard	Captive	E5	Pass	Long-term	Largely microbial; captivity caveat, Section 11
Zebrafish intestinal alkaline phosphatase	A	LPS detoxification at the gut interface	Laboratory	E4	Pass	Long-term	Oral enzyme delivery preclinical
Microbial carboxylesterase, fed to piglets (fumonisin)	B	Mycotoxin, a recognised foodborne hazard	Livestock	E4	Not ranked	—	Exogenous microbial enzyme fed to piglets, not a pig adaptation; Tier B, mechanism only (Section 5)
Geophagia (red deer, parrots)	A	Adsorption of dietary toxins	Wild, observational	E2-E3	Pass	Near-term	Clay binders in clinical use for related indications; E4 for a foodborne toxin exposure
Colonisation resistance (T6SS, bacteriocins, SCFA)	A	Enteric pathogen exclusion	Mixed; gnotobiotic and human	E1 (M4-M5)	Pass	Near-term	Two approved defined live biotherapeutic products
Tibetan pig Lactobacillus and Bifidobacterium	A	Enteric pathogen exclusion	Livestock	E3 (M1)	Not ranked	—	Compositional association only
Teleost gut gamma-delta T cells and sIgT	A	Gut mucosal immunity	Laboratory	E4	Not ranked	—	Informs oral vaccine design
Murine Paneth cell defensins	A	Gut mucosal immunity	Laboratory	E4	Pass	Middle	Conserved node
Pacific oyster (Vibrio splendidus priming)	A	Vibrio is a foodborne genus; oysters a foodborne vehicle	Laboratory	E5	Not ranked	—	Invertebrate priming, not trained immunity (Section 12)
Bat gut microbiota and microbiota-derived isoflavones (daidzein, genistein)	B	Microbiota-derived metabolites associated with tolerance of infection	Laboratory; heterologous transfer to mice	E4 (M3)	Pass	Middle	Transfer experiment, Section 7.1; no foodborne indication tested
Pacific oyster (early-life microbial exposure, Ostreid herpesvirus-1)	A	Oysters are a foodborne vehicle	Laboratory	E5	Not ranked	—	Microbiome-mediated route, distinct from transgenerational priming (Section 7)
Carnivore NOX1 loss	A	Raw meat consumption with bacterial load	Comparative genomics	E4	Pass	Long-term	Tolerance-class mechanism
Urban rats (TLR and CYP2D selection)	A	Salmonella and E. coli plus chemical hazard	Wild	E3	Pass	Near-term	Survivorship bias caveat, Section 4.1
Dairy cow gut microbiome and peripheral immune cell function	A	Enteric infection	Livestock	E3 (M1)	Not ranked	—	Compositional association only
Bats (PYHIN loss, NLRP3, interferon tuning)	B	Inflammasome and interferon regulation relevant to enteric viral immunopathology	Cell lines, few species	E2	Fails G1; redirected	Middle	No bat study has examined enteric viral infection
Bean bug Burkholderia (pesticide degradation)	B	Microbial detoxification of an ingested chemical	Laboratory	E4 (M3-M4)	Not ranked	—	Symbiont transfer experiment
Nasonia vitripennis and Serratia (atrazine)	B	Microbial detoxification of an ingested chemical	Laboratory	E5 (M1)	Not ranked	—	Cited for mechanism only
Ticks and planthoppers (epigenetic detoxification)	B	Epigenetic calibration of detoxification enzymes	Laboratory	E5	Not ranked	—	Cited for mechanism only; own challenge is chemical
Smittia aterrima CYP and GST expansion	B	Xenobiotic handling relevant to ingested chemical hazard	Comparative genomics	E5	Not ranked	—	Cited for the xenobiotic-handling mechanism only
Sahara silver ant heat-shock proteins	B	Proteostasis at epithelial interfaces	Wild and laboratory	E3	Pass	Middle	Counter-case in Section 4.1 (heat stress and Salmonella)
Large yellow croaker HIF-1alpha and TLR	B	Hypoxia-immune crosstalk	Aquaculture	E5	Not ranked	—	Weak foodborne link; retained for pathway only
Janthinobacterium lividum (violacein)	B	Antifungal biocontrol relevant to mycotoxin prevention	Laboratory	E4	Not ranked	—	Food-system application
Purple sea urchin TLR expansion	C	Multiplexed recognition architecture for biosensor design	Comparative genomics	E4 (ex vivo only)	Fails G2 as therapy	Near-term as device	Rejected for host engineering, Section 8.2
Horseshoe crab Factor C (LAL assay)	C	Proof that an invertebrate receptor cascade reaches regulatory standard	Commercial and regulatory	E1	Pass (precedent)	Near-term	Precedent underwriting the biosensor proposal
Pangolin cGAS-STING and exosome delivery	C	Delivery-system concept	Comparative genomics	E5	Pass	Long-term	No mammalian model has tested the proposal
Duffy-null allele; IFNL4	C	Proof that protective human variants are clinically consequential	Human genetics	E1	Not ranked	—	Not foodborne; feasibility only
Avian and mammalian PKR and MX1 positive selection	C	General antiviral evolution	Comparative genomics	E5	Not ranked	—	General antiviral evolution; no foodborne application proposed
Cetacean MX1 loss	Excluded	No identifiable human translational route	Comparative genomics	n/a	Fails G1	Excluded	Retained in Section 8.2 solely as a negative case

Tier B and Tier C exemplars support mechanistic argument or establish feasibility. They are not offered as evidence about foodborne disease resistance.

Tiers are defined in Section 1.2. Evidence levels (E1 to E5) and causal levels for microbiome claims (M1 to M5) are defined in Section 1.3, and the microbiome evidence is discussed further in Section 7.1. Both scales record how far a claim has travelled rather than how large or how certain the effect is. The gate outcome and priority group columns apply the decision rule of Section 1.4; an exemplar marked “Not ranked” supports a mechanism or establishes feasibility rather than entering the ranking as a candidate in its own right.

### 2.3 Classification of evidence

Being on topic is not the same as being well supported. An example can sit squarely inside the scope set above and still rest on thin evidence, so every admitted example is graded next. Every proposal carries a label showing how far it has been tested in people. E1 means already approved or in routine use for this or a closely related purpose. E2 means tested in controlled human trials but not approved for this use. E3 means studied in humans but not in a trial. E4 means shown only in animals, organoids or germ-free systems. E5 means seen only in the original animal, with nothing tested in humans or human models. Proposals at E1 to E3 are written as things that can be developed; those at E4 and E5 are written as open questions that state what has not yet been tested. This scale is ours, and it is not a clinical evidence hierarchy. Systems such as GRADE and the Oxford levels rate how much confidence a trial result deserves (46, 47). Our labels record something different: how far a mechanism has moved toward human use, which is the question a reader of this review needs answered. They align with the T0 to T4 phases of translational research (48), with E4 at T0, E3 at the boundary of T0 and T1, E2 across T1 and T2, and E1 at T3 and T4. We add one level below that scheme. E5 covers findings reported only in the source animal, and keeping it separate from E4 matters here because much of the comparative literature has never been tested in a human or in any mammalian model.

It is worth separating three questions that are easy to run together. How far has a mechanism moved toward human use? That is what the E scale records. How much confidence does a reported result deserve, and how large is the effect? That is the work of GRADE and the Oxford levels, and we do not attempt it here. Does the intervention work against foodborne disease? That is a claim about efficacy, and no level on this scale makes it. A high grade therefore describes the state of development and is not a recommendation. The tier and the grade describe different things and should be read together. A tier describes the animal example and limits what it can be cited for; a grade describes the human proposal drawn from that example and says how far the proposal has travelled. **Table 2** reports both for every example, so a well-developed proposal resting on a Tier B example can be recognised as such. The distinction is sharpest at E1, where an agent may be approved for one purpose while the foodborne indication remains untested; Section 4 gives the case of tapinarof, an approved topical agonist of the aryl hydrocarbon receptor that is still E4 for any foodborne use. Where the two differ we report both and name the indication. The same reading applies at the other end. E5 records that nothing has been tested in a human or a human model, not that the underlying animal work is weak.

**Table 2:** The decision matrix of the biomimetic immunity continuum.

Candidate class (section)	Gates: conservation, tolerability, decomposability	Evidence	Existing agent or route	Risk and reversibility	Scalability	Limiting step	Priority group
Dietary and phytochemical interventions (4, 5)	All pass	E2; E4 for a foodborne indication	Yes: foods and supplements	Low; intermittent and reversible	High	Trial evidence for a foodborne endpoint	Near-term
Clay and mineral binders (5)	All pass	E2-E3 related indications; E4 foodborne	Yes: veterinary and feed-grade	Low; dosing and micronutrient binding	High	Trial evidence for a foodborne exposure	Near-term
Defined probiotic and postbiotic consortia (7)	All pass	E1 and M5 for C. difficile; E4 foodborne	Yes: two approved products	Moderate; colonisation may persist	High	Trial evidence for a foodborne endpoint	Near-term
Biosensor diagnostics (3, 5)	All pass; acts ex vivo	E4; E1 precedent (LAL assay)	Yes: device regulatory route	None; acts outside the body	High	Validation in real food matrices	Near-term
Heat-shock inducers (4)	All pass	E3	Partly: nutraceuticals, geranylgeranylacetone	Moderate; strongly dose-dependent	Moderate	Formulation and dosing for a gut endpoint, with scalability co-limiting	Middle
Epigenetic and metabolite primers (7, 12)	All pass	E4; M4 for butyrate	Partly: fibre, defined consortia	Moderate; a trained state is hard to reverse	Moderate	Delivery and target specificity, with scalability co-limiting	Middle
Engineered antimicrobial peptides (6.1)	All pass	E1 in food preservation; E4 as therapeutics	Partly: food-grade agents exist; no systemic agent	High; narrow therapeutic index, transferable resistance	Moderate	Stability, delivery and cross-resistance, with scalability co-limiting	Middle
Recombinant gut-luminal enzymes (5)	All pass	E2 as molecules; E4-E5 for this use	No: no gastric-stable oral route exists	Low	Moderate	Gastric-stable oral delivery	Long-term
Gut-targeted genome modulation (3)	All pass	E4	No	High; durability of the edit	Low	Targeted *in vivo* delivery, with risk and scalability co-limiting	Long-term
Exosome and miRNA therapies (3, 7)	All pass	E5	No	Not characterised	Low	Enabling delivery technology, with risk and scalability co-limiting	Long-term
Synthetic multiplex receptor arrays (3)	All pass; built as a device	E4	No integrated platform	None in the host	Low	Integrated platform does not yet exist, with scalability co-limiting	Long-term
Gastric acid augmentation (8.2)	Fails tolerability: sustained acidity above the normal human range	n/a	n/a	n/a	n/a	Humans already have a strong acid barrier; further augmentation shows no benefit and adds risk	Excluded; redirected to preserving the barrier
Host receptor-repertoire expansion (8.2)	Fails tolerability	n/a	n/a	n/a	n/a	Expected autoimmunity in a host with adaptive immunity	Excluded; redirected to ex vivo diagnostics
PYHIN gene deletion (8.2)	Fails conservation in context and decomposability	n/a	n/a	n/a	n/a	Loss depends on companion changes humans lack	Excluded; redirected to transient NLRP3 damping
Cetacean MX1 loss (8.2)	Fails conservation	n/a	n/a	n/a	n/a	No identifiable human route	Excluded; retained as a negative case

Scalability combines cost per person, the regulatory route available, and time to implementation. These ratings are qualitative and internal to the framework rather than the output of a health-economic analysis. n/a indicates that a candidate excluded at a gate is not ranked on the remaining criteria.

Three criteria act as gates and remove a candidate from the ranking when failed; four criteria then set the order, evidence carrying the grade assigned in Section 1.3 and the other three recorded on short ordinal scales with the reason shown beside each rating. Candidates are ranked on the limiting step, the weakest of the four in the sense defined in Section 1.4, rather than on an average or a composite score. Evidence levels E1 to E5 and causal levels M1 to M5 are defined in Section 1.3; the gates and the ranking rule are set out in Section 1.4; the four excluded adaptations are discussed in Section 8.2. Where two or more criteria are equally slow they are named together as the limiting step; the key below the table shows the time each rating implies.

**Table:** Key to **Table 2**.

Criterion	Cleared	Near-term	Middle	Long-term
Evidence (Section 1.3)	E1 to E3 for the stated indication	E4	E5	Never from evidence alone
Existing agent or route	Yes: agent and route both exist	—	Partly: an agent exists, but delivery or formulation is unsolved	No: no agent, or the route needs technology not yet in hand
Risk and reversibility	None to moderate	—	High, but stopping the intervention ends the exposure	Irreversible, uncontainable or not characterised
Scalability	High	—	Moderate	Low

How each rating translates into time. A candidate’s group is its slowest time; the limiting step is the criterion, or criteria, at that time. Where a candidate carries two evidence grades, the grade for the foodborne indication is used.

Claims about gut bacteria carry a second label showing how firmly cause has been established. M1 means the bacteria are simply found alongside the effect. M2 means removing the microbes changes the effect. M3 means transferring the microbes into a germ-free animal reproduces it. M4 means one identified microbe, gene or molecule is responsible, shown by removing it or by giving it alone. M5 means a controlled human trial. Words implying cause are used only from M3 upward. This ladder applies the commensal Koch’s postulates, the standard rules for showing that a gut microbe causes an effect, to the comparative setting (49), and the wider problem it addresses, that association in microbiome data is not causation, is set out elsewhere (50). Two limits are worth stating. Transfer into a germ-free animal shows that the microbes are sufficient in that animal, not that they cause the effect in people (51), so M3 is a threshold for careful wording rather than proof. And where an effect belongs to a whole community rather than to one organism, as it does for colonisation resistance, rules written for a single microbe do not apply, and the argument has to be made about the community instead (52).

The M ladder invites the same confusion and needs the same caution. It grades how firmly cause has been established in the system where the work was done. It does not grade the size of the effect, and it says nothing about whether that effect would appear in a person, since M5 is the only level that involves people at all. A claim can sit high on M and low on E, or the reverse, because the two scales answer different questions. Neither judges how well a study was done: a well conducted trial and a poorly conducted one both sit at E2, and a reader who wants that judgement should use the tools built for it. **Supplementary Table S2** sets out, level by level, what each label asserts and what it does not, and **Supplementary Table S3** compares both scales with GRADE, the Oxford levels and the T0 to T4 continuum.

One consequence of these scales is worth stating as a rule, because it governs how every pillar in Sections 3 to 7 is written. We separate two kinds of claim. A mechanistic inspiration is an animal finding that points to a human molecule or pathway worth examining. It is written as a question, it names the system the work was done in, and it carries E4 or E5, or M1 to M4 where the claim concerns gut bacteria. A candidate intervention is a proposal for which an agent, a route into the body and human data already exist. It is written as something that can be developed, and it carries E1 to E3, or M5. The grade is what separates the two, so a reader can tell which kind of claim is being made from the label alone. Several pillars contain both, the antimicrobial peptide and microbiome pillars most obviously, and where they do we mark each proposal rather than letting the stronger cases carry the weaker ones. Nothing at E4 or E5 is offered as an intervention with demonstrated translational potential, however attractive the underlying biology.

### 2.4 How we decided which adaptations are worth pursuing

The tier decides what may be discussed and the grade records how far a claim has travelled. Neither asks the question this section asks, which is whether an adaptation could work in a person at all. Far more animal adaptations have been described than could ever be turned into treatments, and the most eye-catching ones are not always the most useful. We therefore ask the same six questions about every adaptation. First: do humans still have the same molecule or pathway? Second: is there already a drug, a food or a microbe that acts on it? Third: could the human body cope with that change at the strength the animal shows? Fourth: how strong is the evidence behind it (Section 1.3)? Fifth: what kind of risk would we be taking on (Section 1.1)? Sixth: would one purified ingredient reproduce the effect on its own, or is the whole system needed (Section 8.3)? The answers put candidates in order of priority; most of the time a poor answer defers rather than excludes. An adaptation that does poorly on these questions is not abandoned but moved into a slower group, because what usually holds it back is one missing piece of technology: a way to get a drug as far as the gut, a way to stop a peptide being digested before it works, or a way to build a bacterial mixture to a known recipe. Failure on the first, third or sixth question is not recoverable in the same way: these three act as gates, set out in full below, and failing one excludes the candidate outright. Section 8.2 sets out the four adaptations our own criteria reject on these grounds; in three the rejection points the question somewhere else, and in one it ends it.

The six questions do not carry equal weight, and it is worth stating how they combine, because the order in which we place candidates follows from that combination rather than from an overall impression. Three of the questions act as gates; the other three, together with scale, which the ranking has used all along without naming it, set the order. It is worth saying plainly which is which. The first, third and sixth questions are the gates: does the human pathway still exist, could a person tolerate the change, and can a deliverable unit be defined at the level at which the effect works? These are pass-or-fail. The second, fourth and fifth become the ranking criteria: does an agent already exist, how far has the evidence travelled, and what kind of risk would the intervention carry? These are matters of degree. To them we add scale, which asks what an intervention would cost and how long it would take.

The gates are conservation, physiological tolerability and decomposability. A candidate fails the conservation gate if humans no longer carry the molecule or pathway, or carry it only without the companion changes the trait depends on. It fails the tolerability gate if the human body could not sustain the change at the strength the animal shows. The decomposability gate asks whether a translatable unit can be defined at the biological level at which the effect actually operates, and whether that unit can be reproduced, controlled and delivered to a patient. The unit may be a molecule, a single organism or a defined community, and what matters is that it can be specified and supplied rather than how simple it is. A candidate fails the gate when no such unit can be defined at any level, for example when the effect depends on the intact animal or on a state that cannot be reproduced outside it. The causal grade feeds this gate directly. A claim about gut bacteria below M3 has not been shown to depend on one identified microbe or molecule, so it cannot be offered as one; Section 7.2 explains why this often reflects how the biology works rather than a gap in the evidence. Such a claim can still pass the gate as a defined consortium, provided the community can be specified, manufactured and given reproducibly, since the level at which the unit is defined is part of the test and not a concession made afterwards. Failing a gate is not a matter of degree: it removes the candidate from the ranking altogether. This is where the two earlier labels take effect. The tier has already settled what may be discussed, so everything reaching this point is admitted. The evidence grade, by contrast, is not a gate at all: a candidate graded E5 can clear all three gates and simply rank low, because thin evidence is a reason to wait rather than a reason to rule something out. Only the causal grade constrains a gate, and only the third one, for the reason just given. Two examples make the tolerability gate concrete. A sodium-channel mutation lets softshell clams feed on dinoflagellates that carry the toxin saxitoxin, and three substitutions in the sodium pump let monarch caterpillars feed on milkweed (53, 54). Humans carry both proteins, so conservation is not the difficulty. The difficulty is that neither can be altered in a person and survived, and both cases are set out in **Supplementary Table S1**.

Passing the gates means a candidate is possible, not that it is ready. Four things still stand between a promising animal finding and a person being helped by it: whether it has been shown to work in people, whether the tool to do it already exists, whether it would be safe and possible to undo, and whether it could be afforded and delivered at scale. The four criteria are simply those four obstacles, and every candidate that passes the gates is rated on all four. Evidence carries the grade already assigned in Section 1.3 rather than a fresh rating. Accessibility is recorded as yes, partly or no, according to whether a usable agent or route exists. Risk and reversibility, and scalability, are each placed on a short ordinal scale, with the reason for the rating given beside it rather than left implicit. Evidence is nothing new. It is the grade already given in Section 1.3, E1 to E5, together with the causal level M1 to M5 where the claim concerns gut bacteria, and this is the point at which that grade does its work. Accessibility asks whether a drug, a food or a microbe that acts on the human pathway already exists. Risk and reversibility turn the defence class of Section 1.1 into a practical question. Copying resistance drives the pathogen to evolve around the treatment; copying tolerance leaves a person infectious for longer; avoidance carries neither cost. The criterion also asks whether the change can be stopped, or left to fade, once it has been made, and Section 10 sets out why that matters. Scalability asks what it would cost per person, what regulatory route exists, and how long it would take to reach people. These are qualitative judgements made inside the framework, based on the products and routes that exist today. We present no formal health-economic evaluation, so no rating here should be read as a cost-effectiveness estimate or as a validated feasibility score.

The four criteria are not averaged. We rank on the limiting step, which is the weakest of the four in the sense set out below, because that is what determines when a candidate could reach use. Put plainly, a candidate is placed by whatever is holding it back most, since that is the obstacle which has to be cleared before any of the others matter. A proposal can look strong on three counts and still be a decade away because of the fourth, and it is the fourth that decides when it could arrive. Which group a candidate lands in follows from what kind of obstacle the limiting step is, and that mapping is fixed rather than decided case by case. A candidate whose only weakness is missing evidence, where a trial using agents that already exist could supply it, belongs in the near-term group. A candidate whose weakness is formulation, stability or delivery, with the principle already established, belongs in the middle group. A candidate whose weakness is an enabling technology that does not yet exist, or an inability to reverse or contain the intervention once given, belongs in the long-term group. Three cases show the rule at work. Defined microbial consortia already have an agent, a route into the body and an acceptable risk profile; what they lack is a trial against a foodborne endpoint, so evidence is the limiting step and they sit in the near-term group. Engineered antimicrobial peptides rest on an established principle but are digested before they can act, so the limiting step is formulation and they sit in the middle group. Gut-targeted genome modulation waits on enabling technology that is not yet in hand, so it sits in the long-term group whatever the appeal of the underlying biology. Set out as a procedure it is short. Check the three gates, and a failure ends the matter there. Rate the four criteria. Find the weakest. Read off the group that kind of weakness implies. **Table 2** is that procedure carried out for every proposal class in this review: one row for each class, a column for the gates and one for each of the four criteria, a column naming the limiting step and a column giving the resulting group, so the whole judgement for any candidate can be read across a single line. It is the rule that produces the ordering set out in the Summary, and it covers both the proposal classes retained here and the four adaptations the gates exclude.

Ranking on the limiting step needs a rule that anyone can apply, so we set it out here. Each of the four criteria is either cleared or open. Cleared means it will not delay the candidate. Open means work is still needed on it. Every open criterion is then given a time: near-term, middle or long-term. That time is read from the rating in **Table 2**, not decided afresh for each candidate. The key below **Table 2** lists every rating and the time it implies.

The three times have plain meanings. Near-term means the only thing missing is a trial in people, using agents that already exist. Middle means the principle is established but the product is not, so formulation, delivery, dose or manufacture still have to be worked out. Long-term means something has to be invented first, or the change could not be undone or contained if it went wrong. Thin evidence on its own never makes a candidate long-term, because a trial can be run with what exists today.

The group is the slowest of the open criteria, and that criterion is the limiting step. A candidate can look strong on three counts and still wait years for the fourth, so the slowest one decides. If two or three criteria are equally slow, all of them are named and the group stays the same. Nothing has to be broken open, because the group depends on the time and not on which criterion carries it. No criterion outranks another. Each candidate therefore falls into one group only, since only one time can be the slowest. Where a candidate has one rating for general use and another for foodborne disease, the foodborne rating is used.

Two examples show the rule at work. Defined microbial consortia are cleared on agent, risk and scale, and only the foodborne trial is missing, so the trial is the limiting step and they are near-term. Recombinant gut enzymes are cleared on risk, but no form of them survives the stomach, so delivery is the limiting step and they are long-term. Applied to the eleven ranked candidates, the rule gives the group shown in every row of **Table 2**.

Because what comes out of this is a group rather than a number, we should be explicit about what that judgement is and is not. The levels are ranks rather than measurements, and they record our judgement. We deliberately do not convert them into a composite number. A single score would imply a precision the underlying evidence does not support, and it would let a strong rating on one criterion hide a weakness on another that should rule the candidate out, which is exactly what the gates exist to prevent. Ranking on the limiting step is also easier to reproduce than a weighted score, not harder. A weighted score requires agreement on the weights, and the weights are the part of any scoring scheme that is hardest to justify and easiest to dispute. The limiting step needs none: two readers who agree on the four ratings will reach the same group, and two who disagree can see exactly which cell they differ on. What the table offers is a line of reasoning others can repeat, rather than an answer they will necessarily reach: a reader who disagrees with a placement can see which cell the disagreement sits in, change that cell, and follow the same rule to a different conclusion. This gives the hypothesis the review is built on, stated in the form the framework can test. Among adaptations that act on a human pathway which is conserved or otherwise reachable, translational potential depends more on conservation, accessibility, safety and deliverability than on the size of the effect seen in the source animal. The claim is deliberately limited to candidates that pass the gates, since an adaptation that humans cannot host is excluded before any of this applies, and it says that phenotype magnitude adds little to the ordering rather than that striking animals are useless. The endpoint the framework predicts is progression toward use rather than efficacy, and the validation subsection of the Summary sets out how the two are scored apart.

Taken together, Section 1 asks four questions of every animal adaptation in this review, in the order they appear above. What kind of defence is it: avoidance, resistance or tolerance (Section 1.1)? Is it about our problem, meeting one of the three tiers (Section 1.2)? How far has the claim travelled toward human use, and how firmly has cause been established, on the E and M scales (Section 1.3)? And could it work in a person, which is the gates and the ranking set out here? Each question uses the answer to the one before it. The first supplies the vocabulary and the class of risk that the ranking later weighs. The second decides what enters the review and what it may be used to claim. The third records how strong each admitted claim is. The fourth decides what is worth pursuing, and in what order. A candidate can leave at the second question for being off topic, or at the fourth for failing a gate, but never for weak evidence alone, which changes a candidate’s place in the queue rather than its place in the review. **Figure 1** shows the same sequence as a single path, and **Table 2** gives the result for every proposal retained here.

**Figure 1:**
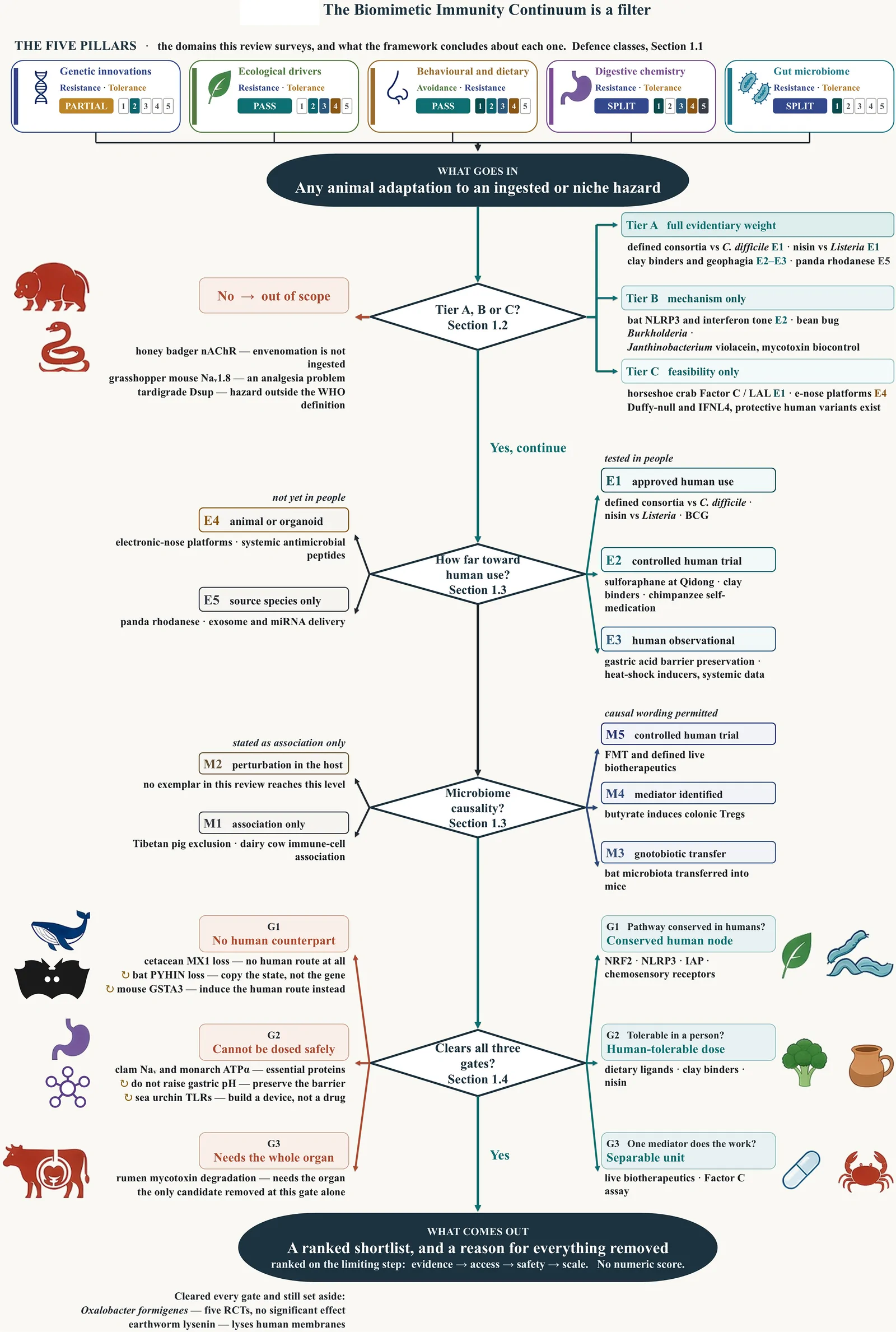
The biomimetic immunity continuum is a filter. The five pillars surveyed in this review are shown at the top with the verdict the framework reaches on each, and every candidate adaptation enters the filter from one of them. A candidate then meets four labels, in the order in which the article introduces them. Defence class (Section 1.1): each pillar is marked avoidance, resistance or tolerance, or a combination of these; resilience is their composite outcome and is never assigned. Inclusion tier (Section 1.2): a candidate is admitted only if it meets one of three tiers, and each tier carries a different licence. Tier A examples carry full evidentiary weight, Tier B examples are cited for mechanism only and never as evidence about foodborne disease, and Tier C examples establish feasibility only and are labelled as such. A candidate meeting none of the three is out of scope. Evidence, E1 to E5 (Section 1.3): how far a proposal has moved toward human use. The branch divides where human evidence begins, since E1 to E3 rest on people while E4 and E5 do not. A grade records how far an application has moved toward human use, not how well it works. Causal strength, M1 to M5 (Section 1.3, applied in Section 7.1): a second label carried only by microbiome claims, recording how firmly cause has been established. The branch divides at M3, the threshold above which causal verbs are used, and claims at M1 and M2 are stated as associations. Transfer into a gnotobiotic animal demonstrates sufficiency in that model rather than causation in people, and where an effect is a property of the community rather than of one organism, the ecological form of the argument replaces the single-microbe postulates. Gates (Section 1.4): admitted candidates must clear three gates. The pathway must be conserved in humans, the intervention must be tolerable in a person, and a single mediator must carry the effect. Survivors are then ranked on the limiting step, evidence then access then safety then scale, with no numeric score, and the priority group each surviving candidate receives is given in **Table 2**. A candidate that clears every gate may still be set aside on evidence or on safety, as shown beside the ranking box. Throughout, the teal spine is the retained path: a candidate moves down it only by passing the step above. Rust marks a candidate removed at that step, and the symbol ↻ marks one redirected rather than rejected. In the pillar cards a filled evidence cell means that the proposal this review advances for that pillar carries that grade; the cells are not a score and are not additive. The two scales are set side by side in **Supplementary Table S2**, per-exemplar tiers, settings and grades in **Table 1**, the five pillars in **Table 3**, and every excluded candidate with the reason for its exclusion in **Supplementary Table S1**. Abbreviations used in the figure: nAChR, nicotinic acetylcholine receptor; ATPα, the alpha-subunit of the Na+/K+-ATPase; LAL, Limulus amoebocyte lysate.

This review is built on a hypothesis, not simply on an aim. For many of these defences humans still carry the pathway and leave it switched off. That is not true of all of them: some adaptations depend on genes, on genomic context or on companion changes humans do not have, and those are removed at the gates in Section 1.4. If that is right, then what makes an animal adaptation useful is whether humans still carry the same pathway and whether a drug or a diet can switch it back on, not how dramatic the animal is. The prediction can turn out to be wrong. It applies to the adaptations that pass those gates, and it says that within that set how striking the animal is adds little once conservation, accessibility, safety and deliverability are known. Most work on copying animals has assumed the reverse and has concentrated on the striking cases, while the mechanisms that actually reached patients have been unremarkable ones. Section 9 tests whether the prediction holds.

### 2.5 How this differs from related fields

Several established fields already cover part of this ground. Evolutionary medicine explains why modern humans fall ill, but it does not say which treatment to try first (55). Ecological immunology explains why wild animals spend different amounts of energy on their immune systems, and it does not set out to produce therapies (56). Trained immunity is a single mechanism rather than a way of organising many, so it appears here as one entry among others (57). Biomimetics supplies the idea of copying nature but no rule for deciding what to copy. The gap they leave is a practical one. Comparative biology now describes new animal adaptations faster than anyone can test them, and the decision about which to pursue is usually made informally, often on the basis of how impressive the animal is. Our framework addresses only that decision. It takes one candidate treatment at a time, puts it in order of priority, says what kind of risk it carries, and can reject it outright.

### 2.6 How the literature was identified

This is a narrative review, not a systematic one. The question spans comparative immunology, microbial ecology and clinical medicine, and the studies differ too much in design to be pooled statistically. We did not attempt a systematic search and we do not report one. What we can state precisely is how each example was judged once it had been found: the inclusion tiers set out in Section 1.2 and the evidence levels set out in Section 1.3 were applied to every example, and both are reported for every example in **Table 1** (45).

Examples were assembled in three ways: from searches of PubMed/MEDLINE, Web of Science and Scopus; from following citations backward and forward out of the papers we found most useful; and from the authors’ own reading in comparative immunology and food safety. Only English-language sources were used. We do not claim the procedure was exhaustive, and a different group of authors would have assembled a partly different set of examples. For each example we recorded the setting in which its evidence was generated: observation of wild animals, field or on-farm study of captive and domestic animals, laboratory experiment including cell and gnotobiotic systems, comparative analysis of genomes, or study in humans. These settings are listed for every example in **Table 1**, and we give most weight to mechanisms where field and experimental evidence agree.

Four limitations follow, and the first matters most. Because the search was not systematic, the choice of examples rests on our judgement, and a reader cannot check that judgement against a record of what was left out; **Table 1** is offered so that the treatment of each example we did include can at least be checked. Well-studied animals, bats above all, appear more often than their share of the relevant biology. Journals publish positive results more readily than negative ones, which compounds the bias described in Section 4.1. And working only in English will have missed work in veterinary and agricultural journals.

## 3 Diversity of foodborne pathogens: categories, molecular mechanisms, severity, and links to animal adaptations

To provide a structured foundation for the translational discussions that follow, we briefly categorize the major foodborne pathogens by mechanism, signalling pathways, and clinical severity, and map them to the most relevant animal adaptation pillars and solutions developed in later sections.

Bacterial pathogens can be subdivided into: (i) Toxin-producing (e.g., Shiga toxin-producing E. coli [STEC], Clostridium perfringens, *Staphylococcus aureus* enterotoxins) that primarily activate NLRP3 inflammasome, ribosomal stress responses, or MAPK pathways, leading to bloody diarrhoea and haemolytic uremic syndrome (HUS, mortality 3–5% in children) (58–60); (ii) Invasive enteropathogens (Salmonella, Campylobacter, Shigella) triggering TLR4/TLR5 and NLRC4 inflammasome with potential for bacteraemia and Guillain-Barré syndrome (GBS, approximately 30 per 100,000 infections) (61–63). Viral agents rely on distinct entry routes: histo-blood group antigens and integrins for norovirus and rotavirus (64), gangliosides for hepatitis A (65), and these agents induce strong IFN and inflammasome responses (66). Parasites (Giardia, Cryptosporidium, Entamoeba) and helminths activate distinct Th2/ILC2 and Treg pathways (67, 68). Mycotoxins (DON, ZEA, fumonisins) from Fusarium/Aspergillus activate MAPK, NRF2, and ribotoxic stress responses (69).

These categories map directly onto animal solutions developed in this review: zebrafish intestinal alkaline phosphatase and panda rhodanese, together with vulture gastric hyperacidity, whose translatable inference is preservation of the barrier rather than its augmentation (Section 8.2), for toxin neutralization and LPS detoxification (7, 70–72); bat interferon priming, which restricts viral replication and is a resistance mechanism, together with PYHIN loss, which caps immunopathology and is a tolerance mechanism (the two are distinguished in Section 3) (73); microbiome competitive exclusion (Type VI secretion systems, bacteriocins, short-chain fatty acids) (74) and trained immunity for bacterial pathogens (Section 12) (75); behavioural self-medication with plant secondary metabolites for gastrointestinal parasites (Section 5); and phytochemical and enzyme detoxification (76), aryl hydrocarbon receptor and KEAP1-NRF2 xenobiotic handling (Section 4), plus geophagia-inspired clay binders for mycotoxins and alkaloids (77). **Table 3** summarises the five pillars, the mechanisms within each and the translational applications proposed for them; Section 6 occupies two rows there because it covers two distinct mechanism classes, extreme digestive chemistry and specialised immune mechanisms. The sections that follow develop these mappings. Each proposal carries the evidence level defined in Section 1.3, from E1, approved human use, to E5, observation in the source species alone. Both labels are read as Section 1.3 defines them, and **Supplementary Tables S2** and **S3** set out what each level asserts and how the two scales relate to the established frameworks.

**Table 3:** Biomimetic strategies for combating foodborne diseases: molecular mechanisms and translational applications.

Animal adaptation category	Key animal examples	Specific molecular mechanism	Potential clinical translation & biomimetic application	References
1. Evolutionary Genetic Innovations (Section 3)	Insects, Purple Sea Urchin, Bats, Pangolins	Pattern Recognition Receptors (PRRs): PGRP-SA and GNBP1 in insects enable rapid detection of bacterial cell wall components. Toll-Like Receptor (TLR) Expansion: 253 TLR paralogues in sea urchins for broad pathogen sensing. Positive Selection: Rapid evolution of PKR and MX1 genes in birds/mammals enhances antiviral interferon response. Strategic Immune Gene Inactivation: Loss of cGAS and STING genes in pangolins dampens inflammatory signalling. Exosome Delivery System: Pangolins employ exosomes for targeted delivery of immune modulators like Lysozyme (LYZ) and S100 proteins	Diagnostics: Engineer biosensors using the diverse ligand recognition of animal TLRs. Immune Engineering: Explore CRISPR-based gene editing to fine-tune human immune responses, inspired by adaptive gene loss. Targeted Anti-inflammatory Therapies: Develop cGAS/STING pathway inhibitors inspired by pangolin gene loss to treat autoimmune and inflammatory diseases. Advanced Drug Delivery: Design synthetic exosome platforms for the targeted delivery of antimicrobials (LYZ) and immune modulators (S100) to specific tissues.	(8, 79, 80, 88, 97, 98, 100, 220)
2. Ecological Drivers (Section 4)	Urban Rats, Insects (*Smittia aterrima*), Large Yellow Croaker	AHR/KEAP1-NRF2 Pathway Activation: Upregulated by PAHs, metabolizes toxins. CYP & GST Expansion: Gene family expansion for detoxification (e.g., in insects). HIF-1α & TLR Reprogramming: Enhanced survival under hypoxia/bacterial stress.	Gene Therapy & Enzyme Inducers: Proposed and untested for a foodborne indication: small molecules to activate AHR/KEAP1-NRF2 pathways for toxin clearance. Use epigenetic modifiers to prime these defensive pathways.	(5, 112, 116, 120, 169)
3. Specialized Immune Mechanisms (Section 6)	Bats, Primates, Teleost Fish, Pacific Oyster, Mice	Antimicrobial Peptides (AMPs): Cathelicidins (bats) and theta-defensins (primates) disrupt microbial membranes. Strategic Gene Loss: Loss of pro-inflammatory PYHIN gene family in bats reduces NLRP3 inflammasome activity. Carnivores (Shrew, cats) lose NADPH Oxidase 1 (NOX1), a loss interpreted as reducing the inflammatory cost of a raw-meat diet Mucosal immunoglobulin T (sIgT): Fish-derived antibody for gut pathogen neutralization. Transgenerational Priming: Epigenetic upregulation of immune genes in offspring (Pacific oyster). Retained as a contrast case in Section 12: an invertebrate mechanism with no mammalian counterpart, listed here as a negative case rather than a translational proposal.	Novel Antibiotics: Develop synthetic AMPs inspired by animal peptides to combat multidrug-resistant pathogens. PYHIN Gene Loss: Natural anti-inflammatory state, Inflammasome inhibitors, targeted biologics NOX1 Gene Loss: Gut inflammation control, NOX1-selective inhibitors, gut-protective agents Mucosal Vaccines: Design vaccines that elicit a robust sIgT-like antibody response on mucosal surfaces. Trained Immunity: β-glucans such as lentinan train mammalian innate immunity within a single lifetime; the effect fades and is not inherited (Section 12). The transgenerational route above yields no human proposal.	(8, 30, 75, 88, 101, 153, 154, 217)
4. Behavioural and Dietary Strategies (Section 5)	Mice, Dogs, Chimpanzees, Giant Panda, Red Deer	Vomeronasal Receptor Fpr-3: Detects bacterial peptides (e.g., MgrB), triggering avoidance. Bitter Taste Receptors (T2Rs): Detect quorum-sensing molecules (e.g., from *P. aeruginosa*). Enzyme Rhodanese: Metabolizes cyanogenic compounds into excretable thiocyanate.	Biomimetic Biosensors: Develop sensors using olfactory receptors for real-time pathogen detection. Phytochemical Therapeutics: Harness plant compounds (e.g., from *Vernonia amygdalina*) that disrupt membranes or quorum sensing. Enzyme Therapies and Binders: Recombinant rhodanese has not been tested in humans or in any mammalian model (E5). Clay binders for dietary toxin adsorption are E2 to E3 for related human indications and E4 for a foodborne exposure.	(70, 77, 139, 142, 143)
5. Extreme Digestive Chemistry (Section 6)	Vultures, Wandering Albatross, Zebrafish, Pigs, Giant Panda	Gastric Hyperacidity (pH ~1): Denatures pathogens and toxins. Intestinal Alkaline Phosphatase (IAP): Detoxifies bacterial Lipopolysaccharide (LPS). Specialized Carboxylesterases: Detoxify mycotoxins (e.g., fumonisins).	Barrier Preservation: Protect the gastric acid barrier already present and treat long-term acid suppression as a modifiable risk. Acid-boosting compounds are rejected in Section 8.2. Enzyme Replacement: Proposed and untested for a foodborne indication: recombinant IAP or carboxylesterases to neutralise specific bacterial and fungal toxins in the gut.	(7, 70, 71, 150, 221)
6. Gut Microbiome Dynamics (Section 7)	Tibetan Pigs, Bean Bug, Dairy Cows, Bats	Type VI Secretion System (T6SS): Used by *B. fragilis* to kill competing pathogens. Short-Chain Fatty Acids (SCFAs): Butyrate acts as an HDAC inhibitor, promoting Treg differentiation and tolerance. Microbial Bile Acid Metabolism: Secondary bile acids modulate host immune responses.	Engineered probiotic consortia: Engineer probiotic strains equipped with competitive systems like T6SS. Postbiotic Therapies: Use purified butyrate or other SCFAs as supplements to reduce inflammation and strengthen the gut barrier. Microbiome Modulation: Develop diets or supplements that shift bile acid metabolism to a protective state.	(74, 163, 164, 176)

Entries in the translation column are proposals rather than demonstrated outcomes, and the evidence level for each is given in the text and in **Table 1**.

## 4 Evolutionary genetic innovations in animal immunity and application against foodborne pathogens

Human foodborne disease burden reflects the scale of modern food systems and an immune system calibrated to a different microbial environment, rather than a deficit in immune machinery (78). The adaptations described in this section enhance pathogen detection, inflammation control, immune gene evolution and selective gene loss. This section spans both mechanism classes defined in Section 1.1: pattern-recognition receptor expansion, effector-triggered immunity and antimicrobial peptides are resistance mechanisms, whereas PYHIN loss and NOX1 loss are tolerance mechanisms; bat interferon tuning restricts replication and belongs with the resistance mechanisms. They carry different translational implications and are treated separately below.

One major benefit is improved pathogen recognition (79). Insects have Pattern Recognition Receptors (PRRs) such as Peptidoglycan Recognition Protein SA (PGRP-SA) and Gram-Negative Binding Protein 1 (GNBP1) that detect bacterial components (80). This detection activates immune responses more rapidly (81). The purple sea urchin has expanded its Toll-Like Receptor (TLR) families, featuring 253 paralogues (Tier C; see Section 8.2) (82). This expansion provides broad-spectrum microbial surveillance (Tier C). Effector-Triggered Immunity in metazoans enhances pathogen detection further (4). Murine NLRP1B and NLRP3 sensors detect toxins rapidly and initiate defence mechanisms (83, 84). The nematode Caenorhabditis elegans employs a chemosensory G protein-coupled receptor (GPCR) network to detect harmful bacteria in food and avoid them (85).

Some lineages also limit the cost of infection through changes in inflammatory signalling and immune balance. Bats are one of the multi-pillar models considered here. They are cited for mechanism only (Tier B): no bat study has examined enteric viral or bacterial infection, and the extension to foodborne disease is stated below as an inference rather than assumed. PYHIN loss and attenuated NLRP3 signalling are treated separately below. Recent studies have begun exploring bat ASC2, an inflammasome-suppressing adaptor (86), or chimeric immune modulators that show promise in reducing excessive inflammation in human macrophages and mouse models of viral and bacterial challenge without compromising pathogen clearance (86). Furthermore, the extreme metabolic demands of flight have co-evolved with robust proteostatic mechanisms, including upregulated heat shock proteins (HSPs) that not only protect against protein misfolding under thermal/oxidative stress but may cross-protect gut epithelial integrity during toxin exposure or inflammation (87). These integrated genetic, immunological, metabolic, and physiological traits make bats a multi-pillar case for biomimetic strategies that go beyond single-target interventions (8, 88).

Bats are the exemplar treated at greatest length here, and the one most often misread: their defence against viral infection combines two mechanistically distinct strategies often reported as one.

The resistance arm restricts replication. The type I interferon locus is contracted in *Pteropus alecto*, yet IFN-alpha is expressed constitutively rather than only on induction, and elevated baseline interferon-stimulated gene expression has been described in several species. The result is a cell antivirally primed before infection begins, which lowers viral burden (89). This is resistance as defined in Section 1.1, not a mechanism of reduced inflammation.

The tolerance arm limits damage. The PYHIN family, including AIM2 and IFI16, is absent in several bat lineages, removing the AIM2 inflammasome (90). Separately, NLRP3-mediated inflammation is attenuated through reduced transcriptional priming and a bat-specific splice variant; the two are distinct findings with distinct mechanisms and are frequently conflated (90–92). Dampened STING signalling is also reported. None lowers viral burden; each reduces the cost of carrying it.

The phenotype is the conjunction: replication constrained early by a primed interferon state, immunopathology capped throughout by attenuated inflammasome signalling. The two arms imply opposite interventions with opposite timing. Resistance-mimicking approaches lower viral load and transmission, select on the pathogen, and suit early infection; tolerance-mimicking approaches reduce immunopathology, impose no selection, risk prolonged carriage, and suit established disease. In COVID-19, dexamethasone reduced mortality in patients receiving respiratory support but not in those who were not, the expected behaviour of a tolerance-directed intervention applied across disease stages (93).

Four qualifications apply. Mechanistic data derive from few laboratory-tractable species yet are generalised across an order of more than a thousand species, and whether disease tolerance is a general chiropteran strategy remains open (94, 95). High viral load without pathology is established for few host-virus pairs, and where burden is low the mechanism is resistance. Bats are not uniformly resilient: white-nose syndrome has caused mass mortality and lyssaviruses cause fatal disease. And the bat literature is overwhelmingly viral and largely respiratory; its extension to enteric bacterial and toxin-mediated disease is an inference we state rather than assume.

Pangolins have inactivated innate immune genes like cyclic GMP-AMP synthase (cGAS) and Stimulator of interferon genes (STING) while developing an exosome delivery system (96). This system contains immune modulators like Lysozyme (LYZ) and S100 Calcium-Binding Proteins (97).

Rapid evolution of immune genes is crucial for defending against pathogens (25). Birds and mammals show positive selection of antiviral genes like Protein Kinase R (PKR) and Myxovirus resistance gene 1 (MX1), improving interferon responses against viruses that can be transmitted from animals to humans, including foodborne viruses (98). Cetaceans face an arms race with their TLRs, which change quickly to detect aquatic pathogens (99). Gene loss can also tune immune responses: toothed whales have lost the antiviral gene MX1, a case retained in Section 8.2 as one for which no human translational route is identifiable (100). Similarly, carnivores lose NADPH Oxidase 1 (NOX1), a loss interpreted as reducing the inflammatory cost of a raw-meat diet (101).

A critical consideration for translation is the distinction between deeply conserved and lineage-specific adaptations (102). Core defence modules (TLR and NLR signalling, AHR-KEAP1-NRF2 detoxification, inflammasome regulation, SCFA-GPCR axes, and epigenetic trained-immunity machinery) are ancient and shared across most metazoans, including humans. Humans therefore retain the genetic and cellular toolkit; the opportunity lies in context-appropriate pharmacological, nutritional, or transient genetic activation of these pathways (many agonists/antagonists already exist or are in clinical pipelines) (57, 102, 103). In contrast, certain innovations are lineage-restricted (e.g., PYHIN family loss in several bat lineages, massive TLR paralogue expansion in sea urchins, or microbial fumonisin-hydrolysing carboxylesterases used as a feed additive in pigs) (73, 82, 104). For these, direct transfer is impossible, but they inspire analogous engineering in humans, for example synthetic multiplex PRR arrays or exosome-based delivery of missing modulators. Thus, the Biomimetic Immunity Continuum prioritizes leveraging conserved, druggable nodes for near-term impact while using species-specific examples as blueprints for synthetic or cell-based therapies.

### 4.1 Clinical translation of animal genetic adaptations for foodborne pathogen resistance

The genetic innovations described above suggest routes for clinical translation to enhance human food safety and combat foodborne diseases. How far each of these adaptations can be developed into a therapeutic or preventive approach differs by candidate, and the evidence level attached to each proposal states how far that has gone. Throughout this section and subsequent translation subsections, we explicitly distinguish established animal mechanisms from emerging preclinical concepts and hypothetical future applications. We also specify primary cellular targets and key outcome measures for each proposed strategy in **Table 4**.

**Table 4:** Primary cellular targets and key outcome measures for every strategy proposed in the clinical translation subsections, grouped by pillar.

1. Genetic adaptations		
Strategy	Primary targets	Key outcome measures
Enhanced Pathogen Detection Systems	Gut epithelial cells, resident macrophages/DCs, food-chain monitoring systems	Reduced time-to-detection, multiplex pathogen coverage, lower false negatives vs culture/PCR
Precision Immunomodulation	Innate immune cells (macrophages, monocytes), gut epithelial cells	Reduced excessive cytokine release (IL-1β, IL-6, TNF), preserved pathogen clearance, lower immunopathology
Genome Editing Applications	Gut epithelial cells and lamina propria innate immune cells (transient delivery)	Reduced collateral tissue damage, maintained barrier integrity (ZO-1, claudins), decreased bacterial translocation
Effector-Triggered Immunity	Innate immune sensors in gut epithelium and macrophages; therapeutic exosomes	Rapid priming of innate responses, reduced toxin-induced damage, targeted antimicrobial delivery
Evolutionary-Informed Immune Enhancement	Systemic innate immunity and mucosal surfaces (trained immunity/vaccine responses)	Enhanced heterologous protection, increased vaccine efficacy, measurable trained immunity markers (epigenetic changes in PBMCs)

2. Ecological adaptations		
Strategy	Primary targets	Key outcome measures
Biomimetic Detoxification (AHR Modulators)	Gut and liver epithelial cells, innate immune cells	Upregulated CYP enzymes, reduced toxin bioavailability, attenuated inflammatory storms
Epigenetic Programming	Innate immune cells (monocytes, macrophages, DCs)	Trained immunity markers (H3K4me3/H3K27ac), heterologous protection, reversible epigenetic changes
Thermotolerance and HSP Inducers	Gut epithelial cells, systemic proteostasis	Preserved epithelial barrier during inflammation/fever, reduced tissue damage, systemic benefits
Cross-Resistance Signaling Pathways	HIF-1α/NRF2 pathways in gut/liver/immune cells	Enhanced survival under combined hypoxia/infection stress, boosted immunity in vulnerable patients

3. Behavioural & Dietary strategies		
Strategy	Primary targets	Key outcome measures
Bio-Inspired Pathogen Detection Platforms	Food processing/monitoring systems and consumer-level sensors	Real-time VOC or quorum-sensing detection, reduced outbreak incidence
Phytochemical-Based Interventions	Gut lumen (toxin binding/neutralization), gut epithelium and microbiome	Reduced pathogen/toxin load, preserved microbiome diversity, lower inflammation
Geophagia-Inspired Detoxification	Gut lumen (toxin adsorption), gut barrier	Reduced toxin bioavailability, lower inflammation, improved barrier markers

4. Digestive chemistry & Specialized immune Mechanisms		
Strategy	Primary targets	Key outcome measures
Gastric Acid Modulation	Gastric and upper intestinal lumen, beneficial microbiota	Reduced viable pathogen load, preserved beneficial taxa, lower infection incidence
Antimicrobial Peptide (AMP) Therapeutics	Microbial membranes in gut lumen/mucosa; mucosal innate and adaptive immunity	Broad-spectrum antimicrobial activity with low cytotoxicity, reduced pathogen colonization, enhanced mucosal IgA
Transgenerational Immunity	Innate immune cells and epigenetic machinery (reversible)	Trained innate immunity markers, enhanced heterologous protection without permanent genetic change

5. Host-microbiome synergy		
Strategy	Primary targets	Key outcome measures
Advanced Probiotic Consortia	Microbiome composition and function, gut barrier, innate/adaptive immune tone	Competitive exclusion of pathogens, increased SCFA production, Treg induction, microbiome resilience
Metabolite-Based Immunomodulation	Treg differentiation, gut barrier, AHR pathways in epithelium/immune cells	Increased IL-10/Tregs, strengthened barrier, dual toxin clearance and immune regulation
Cross-Kingdom Signaling	Microbiome composition via miRNA or host signals, early-life immune development	Lasting trained-immunity protection, fine-tuned microbiome-immune tone
Bile Acid Metabolism	Microbial bile acid metabolism, enteric immune responses	Protective secondary bile acid profiles, enhanced enteric resistance with minimal immunopathology

Inclusion here records that a target and an outcome measure can be specified, not that the strategy has been tested in people; the evidence level for each is given in **Table 1**.

#### 4.1.1 Enhanced pathogen detection

Enhanced pathogen detection systems primarily target innate immunity through pattern recognition receptors. Precision Immunomodulation and Effector-Triggered Immunity mainly engage innate immunity via inflammasome control and rapid sensor activation. Genome Editing reduces inflammation through innate pathways, while Evolutionary-Informed Immune Enhancement supports both innate trained immunity and adaptive vaccine responses.

Building on animal models of pathogen recognition, whether detection technologies can be built on the same principle is the question posed here (E4; the Limulus Factor C assay is the E1 precedent). Inspired by the purple sea urchin’s expanded TLR repertoire (253 paralogues), multiplex biosensor arrays capable of detecting multiple foodborne pathogens through pattern recognition receptor technology are put forward here as an ex vivo target rather than a demonstrated one (E4). A clinically and industrially validated example is the Limulus Amebocyte Lysate (LAL) assay, derived from the horseshoe crab’s Factor C serine protease cascade, an ancient arthropod PRR-based innate immune pathway that detects picogram levels of LPS/endotoxin. LAL remains the gold standard for pharmaceutical release testing and is increasingly adapted for food safety screening of Gram-negative contamination (105). Building on this evolutionary success, multiplexed diagnostics could integrate the expanded TLR repertoire of echinoderms with insect PGRP-SA/GNBP1 specificity for bacterial cell wall components and C. elegans chemosensory GPCR networks for quorum-sensing molecules and virulence peptides (E4; the component receptors are characterised, the integrated platform is not). Hybrid electronic nose-PRR biosensor platforms are in prototype development. This is an emerging translational opportunity, building upon the established LAL assay technology, but still requires robust validation in real-world food processing environments.

#### 4.1.2 Precision immunomodulation and anti-inflammatory strategies

Animal genetic adaptations offer novel inflammation-control approaches. Building on bats’ loss of pro-inflammatory PYHIN genes, small-molecule and RNA-based agents that selectively dampen NLRP3 inflammasome activation are in clinical development (E2). Whether damping this pathway would limit excessive inflammation during foodborne infection without impairing pathogen clearance has not been tested, and is stated here as a question. Learning from bats’ unique interferon response, whether targeted IFN modulators could enhance viral clearance without immunopathology is an inference from bat biology rather than a tested human proposal (E4). The human IFNL4 pseudogenizing allele, shaped by ancient adaptation, aids hepatitis C clearance (106) and demonstrates the clinical relevance of fine-tuning interferon pathways. Direct translation of bat PYHIN loss remains speculative. However, early preclinical studies on bat-inspired recombinant IFN modulators have reduced excessive inflammation in human cell lines and mouse models. This is currently an emerging concept requiring validation in humanized systems.

#### 4.1.3 Genome editing applications

Animal evolutionary patterns suggest innovative genetic interventions. Based on the convergent loss of NOX1 in carnivores, whether CRISPR-based approaches could temporarily silence pro-inflammatory genes in gut epithelial cells during foodborne infection is untested beyond organoid and gnotobiotic models (E4); reduced collateral tissue damage would be the intended effect rather than an observed one. What the preclinical work supports is delivery rather than benefit: studies demonstrate nanoparticle or lipid nanoparticle delivery of CRISPR components or mRNA-encoded base editors to intestinal epithelium in organoid and gnotobiotic mouse models, achieving efficient, transient gene modulation without germline alteration. While conceptually promising, *in vivo* genome editing for this purpose remains speculative. Significant technical, regulatory, and safety hurdles exist. These should be considered long-term research goals rather than near-term clinical options.

#### 4.1.4 Effector-triggered immunity and rapid response systems

Animal immune mechanisms provide templates for therapeutics. Whether small-molecule activators of NLRP1B and NLRP3 sensors could provide rapid innate immune priming against bacterial toxins is untested (E5). Inspired by the pangolin exosome delivery of immune modulators (LYZ, S100 proteins), whether therapeutic exosomes carrying antimicrobial peptides could be delivered to the gastrointestinal tract is unknown, and no mammalian model has tested it (E5). Rigorous testing in human organoids and gnotobiotic models is required before clinical consideration can be justified.

#### 4.1.5 Evolutionary-informed immune enhancement

Genetic patterns from diverse species suggest practical applications. Building on positive selection of antiviral genes in birds and mammals, gene therapy to enhance interferon responses against foodborne viruses remains untested in humans (E5). The translational potential of protective genetics is powerfully illustrated by the Duffy-null allele, driven to high frequency in Africa by selection on standing variation, which confers resistance to Plasmodium vivax (107). Nutritional strategies that optimise gut microbiome interactions within human genetic constraints have human mechanistic support (E3). Beyond theory, innate immunity reprogramming is clinical reality: BCG has non-specific effects on heterologous infection in randomised trials (75, 108), while lentinan (β-glucan) is an approved immunotherapeutic adjuvant in Asia (109).

Note on evidence levels: BCG and lentinan are in approved human use (E1), whereas CRISPR-based gut editing (E4) and exosome delivery (E5) have not been tested for any foodborne indication in humans.

## 5 Ecological drivers of foodborne pathogen tolerance and detoxification in animals and application

Emerging evidence shows that strong ecological pressures, including pollution, climate extremes, and agricultural chemicals, are associated with complex molecular adaptations in surviving populations (110). In the specific contexts studied, these adaptations reduce either the burden or the cost of ingested hazards; Section 8.4 explains why this is not a general claim of superior defence. Improvements in detoxification, immune gene evolution, and microbiome-mediated resistance] offer a model for studying foodborne disease and human defence (98, 111). For example, human-made pollution (e.g., car exhaust, PM2.5) activates detoxification systems like aryl hydrocarbon receptor (AHR) and Kelch-like ECH-associated protein - nuclear factor erythroid 2–related factor 2 (KEAP1-NRF2). These systems help metabolize toxins into forms that can be excreted (112, 113). Chronic exposure to polycyclic aromatic hydrocarbons (PAHs) significantly increases the levels of these enzymes through AHR (114). Some species, such as urban rats, improve their resistance to both toxins and foodborne pathogens, like *Salmonella* and *E. coli*, through quick immune gene selection (for example, TLRs) and detoxification genes like cytochrome P450, family 2, subfamily d, polypeptide 1 (CYP2D) and AHR (5). Likewise, Smittia aterrima shows expanded CYP and Glutathione S-transferase (GST) gene families, associated with the capacity to detoxify a wide range of ingested pollutants. The challenge here is chemical rather than microbial, so the case is cited for the xenobiotic-handling mechanism only (Tier B) (111).

Additionally, extreme conditions favour adaptations for protein stabilisation, as in tardigrades, which use intrinsically disordered proteins to survive desiccation (115), and in some species stress tolerance overlaps with immune function. The large yellow croaker (*Larimichthys crocea*) shows better survival rates during bacterial infections under low oxygen levels, an association accompanied by differential TLR signalling and Hypoxia inducible factor 1 subunit alpha (HIF-1α)-mediated reprogramming (116). Similarly, species like the Sahara silver ant (*Cataglyphis bombycina*) produce heat shock proteins (HSPs) that protect against protein damage, a thermal adaptation (117, 118).

Furthermore, the epigenetic mechanisms found in animals, such as DNA methylation, enable the rapid inheritance of resistance traits; the arthropod examples below are cited for mechanism only (Tier B), since their own challenge is chemical rather than foodborne (119). For instance, ticks and planthoppers trigger detox enzymes such as GSTs and CYPs, which confer cross-tolerance between insecticides and thermal stress (120, 121). Whether these mechanisms could be induced in humans to strengthen defence against foodborne disease and environmental toxins is untested and is raised here as a question.

Epigenetic reprogramming of innate immune cells is the other route by which ingested and environmental exposures leave a lasting mark. The two best-supported cases are treated elsewhere: microbiota-derived short-chain fatty acids and DNA methylation at immune gene promoters in Section 7, where their causal evidence is graded, and trained immunity in Section 12, where its duration, context dependence and risks are set out.

### 5.1 Environmental pressure does not reliably enhance defence

Pressure from the environment does not reliably make an animal better defended. What it produces is differential survival: some populations persist, others sicken, decline or die out, and better defence is only one of those outcomes. Selection acts on whatever variation happens to be present and is measured by reproductive success in one particular environment. It does not aim at health and it does not accumulate improvements in a direction. This matters for a review built on comparative evidence, because that evidence comes from the lineages that survived. Populations that failed under the same pressures cannot be sampled, so the literature over-represents successful adaptation and cannot say how often it occurs. When we cite urban rodents as evidence of selection on immune and detoxification genes, we are looking only at the survivors. We treat this survivorship bias as a limitation of our own synthesis as much as of the field.

The relationship between exposure and protection is also not a straight line. Brief or low-dose exposure often induces a protective response, whereas the same exposure sustained, or at high dose, damages tissue, drives chronic inflammation and suppresses immunity. Toxicology has described this two-phase pattern for decades, and it explains how a single mechanism can appear in this review as both a protective adaptation and a hazard (122). The proposals here that work by switching such a pathway on, namely aryl hydrocarbon receptor and NRF2 modulation, heat-shock induction and innate immune training, are therefore specified as intermittent, reversible and dosed within the protective range. Proposals that do not act through these pathways, such as engineered peptides, biosensors and defined microbial consortia, carry different risks and are treated in Sections 6.1, 8.2 and 10.

Four cases in this review cut against our own proposals, and we set them out here rather than leave them to be found. Aryl hydrocarbon receptor activation, which we propose as a protective measure, is also the route by which dioxin causes immunotoxicity, including thymic atrophy and suppressed antibody responses; ligand, dose and duration decide which outcome follows (123, 124). Heat-shock responses protect protein folding, yet heat stress in broiler chickens damages intestinal integrity and increases Salmonella invasion, in the production system from which much human foodborne Salmonella derives (125). Persistent organic pollutants in marine mammals are associated with immunosuppression and death from infection, including the unusually high polychlorinated biphenyl burdens recorded in striped dolphins during the Mediterranean morbillivirus epizootic (126). And successful adaptation by an animal does not translate into reduced risk for people: the urban rodents we cite as evidence of immune and detoxification selection are the same populations that carry Salmonella and E. coli (5). Adaptation by the animal and safety for humans are distinct outcomes, and here they diverge.

### 5.2 Potential clinical translation of animal environmental and ecological adaptations against foodborne pathogens

The molecular adaptations evolved by animals in response to ecological pressures provide a set of candidate strategies, of varying maturity, for human application. Key translational avenues include:

#### 5.2.1 Biomimetic detoxification

Biomimetic detoxification acts on xenobiotic handling through the AHR and KEAP1-NRF2 pathways rather than on immune effector function. Epigenetic Programming acts on innate immune gene expression. Thermotolerance and HSP Inducers strengthen innate barrier protection, while Cross-Resistance Signalling boosts innate immunity under stress conditions.

The robust activation of AHR and KEAP1-NRF2 pathways in pollution-adapted animals provides a blueprint. Instead of chronic toxin exposure, whether pharmacological AHR modulators could pre-activate these defence systems as controlled pre-exposure, priming liver and gut epithelium to metabolise foodborne toxins, is untested for any foodborne indication. Selective AHR agonists from cruciferous indoles could upregulate CYP enzymes, increasing urinary conjugate excretion and phase II enzyme induction; antagonists could dampen acute inflammatory storms. This is pre-induction of a xenobiotic response rather than immunisation: no antigen is involved and no immunological memory is generated. Two safety considerations constrain the approach. The aryl hydrocarbon receptor is the dioxin receptor; sustained agonism is associated with tumorigenic risk in preclinical safety assessment, and CYP1 induction bioactivates several procarcinogens as well as clearing toxins, so the pathway can increase the genotoxicity of some substrates while reducing the burden of others (127, 128). Sustained pharmacological agonism is therefore not a defensible strategy. Intermittent, substrate-aware dietary modulation is supported at E2 for dietary glucosinolates, whereas pharmacological agonists are E1 as a molecule class, tapinarof being an approved topical AHR agonist, but E4 for any foodborne indication. The relevant measurable outcomes are urinary conjugate excretion and phase II enzyme induction markers. Sulforaphane from broccoli, the dietary NRF2 activator, is where that E2 grade comes from. Human evidence for this arm reaches the level of controlled trials: randomized controlled trials of broccoli sprout beverage conducted in Qidong, China produced dose-dependent increases in urinary excretion of conjugates of the airborne pollutant benzene and altered urinary aflatoxin-DNA adducts and phenanthrene tetraols. Aflatoxin is a foodborne mycotoxin and the mechanism is the one proposed here (129–131). The proposal is to induce the human pathway rather than to supply the animal enzyme, and that is a considered choice. Mice tolerate aflatoxin B1 largely because of a constitutively expressed glutathione S-transferase whose activity toward the 8,9-epoxide has no counterpart in human or non-human primate enzymes (132, 133). There is no human protein of that kind to supply or to switch on, which is why the tractable target is the upstream regulator (**Supplementary Table S1**). We note the countervailing consideration that sustained NRF2 activation confers chemoresistance on tumour cells, so the defensible proposal is intermittent dietary-level exposure (134, 135) rather than chronic pharmacological activation, and it is contraindicated in patients receiving cytotoxic therapy (136).

#### 5.2.2 Epigenetic programming for enhanced innate immunity

Induced detoxification enzymes in ticks and planthoppers show that chemical exposure can durably reset the expression of protective genes. Whether small molecules or dietary components could reversibly modulate immune-cell epigenomes against foodborne pathogens is untested in humans (E5). The better-supported epigenetic routes are microbiota-derived metabolites (Section 7) and trained immunity (Section 12).

#### 5.2.3 Thermotolerance and HSP inducers as adjunctive therapy

Learning from the Sahara silver ant, whether heat shock protein (HSP) induction could protect the human gut is the question posed here. Whether HSP inducers (nutraceuticals, mild thermal stress, or agents like geranylgeranylacetone) could protect gut integrity, stabilise epithelial proteins during inflammation or fever, and reduce tissue damage has not been tested against any foodborne indication (E4). Extending this principle systemically, mild thermal stress shows broad promise. For instance, sauna bathing is associated with lower cardiovascular mortality and with a lower incidence of dementia (137, 138); the mechanism is unproven, no heat-shock protein was measured in those cohorts, and HSP induction is one hypothesis among several. This remains an emerging translational strategy. While mild thermal stress and HSP inducers such as geranylgeranylacetone have human observational data for systemic outcomes (E3), their specific application for protecting gut epithelial integrity during foodborne infections rests on preclinical and mechanistic work alone (E4) and requires targeted clinical validation.

#### 5.2.4 Cross-resistance signalling pathways as drug targets

Large yellow croaker HIF-1α–TLR synergy reveals convergent nodes; whether HIF-1α/NRF2 activators could strengthen defence in vulnerable patients against opportunistic foodborne infection is untested. This represents an emerging concept with clear mechanistic rationale. HIF-1α/NRF2 activators are already under investigation in other inflammatory contexts; however, their use to enhance resistance to foodborne pathogens in vulnerable populations remains at the preclinical-to-early translational stage and will need well-designed human studies to establish efficacy and safety.

## 6 Behavioural and dietary strategies for foodborne pathogen avoidance and detoxification in animals

Animals have evolved behavioural and dietary strategies against foodborne hazards that humans largely do not use (139). These mechanisms, which work through specialized sensory-immune systems and bioactive compounds, suggest testable approaches for human food safety.

A key part of this defence is the innate sensory detection of threats in food. Rodents, for instance, use vomeronasal receptors like formyl peptide receptor 3 (Fpr3) to identify bacterial virulence peptides. This detection triggers quick avoidance behaviours (139). Similarly, dogs can find microbial contaminants in food by sensing volatile organic compounds (VOCs), serving as a model for real-time food pathogen detection; the second of these studies concerns disease-associated volatiles in clinical samples rather than in food (140, 141). Mice can detect quorum-sensing molecules from *Pseudomonas aeruginosa* through bitter taste receptors (T2Rs), activating an epithelial innate response; that work was done in nasal rather than gut epithelium, so the case is cited for the receptor mechanism only (Tier C) (142).

Beyond avoidance, animals also self-medicate using plant secondary metabolites (PSMs). Chimpanzees, for example, chew the pith of Vernonia amygdalina, which is associated with reduced nematode burden (143, 144). Herbivores like lambs eat condensed-tannin forages such as Sericea lespedeza, which reduce faecal egg counts (145). The giant panda converts cyanogenic compounds from bamboo into excretable thiocyanate, a reaction attributed to rhodanese activity, though the gut microbiota contributes substantially (Section 11) (70). Similarly, zebrafish utilize intestinal alkaline phosphatase (IAP) to detoxify bacterial lipopolysaccharides (LPS) (72). Microbial fumonisin carboxylesterases, given to piglets as a feed additive, reduce the toxicity of fumonisins, a recognised foodborne mycotoxin (Tier B; the enzyme is microbial, not porcine) (104).

Geophagia, or eating soil, is another detoxification method; red deer consume clay-rich soils that adsorb toxins, bacterial endotoxins, and alkaloids (77). Parrots consume clay at riverbank licks, for which toxin adsorption and sodium supplementation remain competing explanations (146).

Note on evidence levels: geophagia and plant self-medication are well documented in the ethological and ethnoveterinary literature, but that documentation concerns the animal rather than the human application. Phytochemical antimicrobials have reached controlled human trials, but the trial effect demonstrated (berberine in enterotoxigenic E. coli diarrhoea) is antisecretory rather than bactericidal, so E2 attaches to the clinical use and E4 to the membrane-disrupting mechanism proposed here (147)**;** the enzyme-based proposals, giant panda rhodanese and recombinant alkaline phosphatase, are graded under III below (148).

### 6.1 Clinical translation of animal behavioural and dietary strategies for foodborne pathogen defence

These behavioural and dietary strategies translate into three clinical routes, and they do not all act on the immune system. Bio-inspired detection platforms operate before ingestion and are avoidance in the terms of Section 1.1. Phytochemical interventions act on the pathogen itself, principally by permeabilising the bacterial membrane and by interfering with virulence signalling. Geophagia-inspired binders remove the toxin from the gut lumen; under the chemical-hazard convention set out in Section 1.1 this counts as resistance, because it reduces how much toxin is present, and not as enhancement of innate immunity.

These natural strategies, backed by traditional ethnoveterinary knowledge, show the effectiveness of plant-based antimicrobial and anthelmintic compounds. For instance, extracts from *Maesa lanceolata* and *Bolusanthus* speciosus inhibit *Bacillus anthracis Sterne in vitro* and suppress egg hatching and larval development in the nematode *Haemonchus contortus* (149, 150), suggesting leads for natural antimicrobial development. The nematode target here is a livestock parasite rather than a human foodborne pathogen, so only the antibacterial arm bears on the scope of this review.

#### 6.1.1 Bio-inspired pathogen detection platforms

Mimicking canine olfaction, electronic nose devices could detect pathogen-specific VOCs for real-time monitoring. Rodent Fpr3 detection of bacterial virulence peptides and murine T2R detection of quorum-sensing molecules are the two receptor templates; biosensors built on them could flag bacterial signalling and early biofilm formation. The component receptors are characterised and electronic-nose prototypes exist, but no integrated platform has been validated in a food matrix (E4).

#### 6.1.2 Phytochemical-based interventions and engineered detoxification

##### 6.1.2.1 Phyto-therapeutic antimicrobials

Inspired by chimpanzee use of Vernonia amygdalina, plant secondary metabolites are considered here as candidates for natural food additives or supplements, at the maturities given below. Carvacrol permeabilises the bacterial membrane, while chalcone interferes with quorum sensing and toxin production; both reduce virulence gene expression and biofilm formation in foodborne pathogens (76). Activity is established *in vitro* and in food systems. One member of the class has controlled human trial evidence against a foodborne pathogen: berberine shortens enterotoxigenic *E. coli* diarrhoea (E2). That effect is antisecretory rather than bactericidal, so the mechanism proposed here remains untested in people (E4) (147).

#### 6.1.3 Enzyme-based biocontrol and neutralization

Animal adaptations provide blueprints for direct enzymatic neutralization of dietary toxins. Whether a recombinant supplement modelled on giant panda rhodanese could work in the human gut has not been tested in humans or in any mammalian model (E5). Recombinant human alkaline phosphatase is further along: it has reached a phase 3 randomised trial, given intravenously in sepsis-associated acute kidney injury, so the molecule itself is not untested in people; what has not been tried is oral, gut-luminal delivery against a foodborne toxin or endotoxin load (E4) (148). Gastric stability, delivery to the site of action and regulatory classification, rather than the underlying animal biology, are the limiting steps.

#### 6.1.4 Host-detoxification pathway primers

The human route here is dietary, sulforaphane from cruciferous vegetables, but the adaptation being translated is xenobiotic handling through the aryl hydrocarbon receptor and KEAP1-NRF2. That is the pillar developed in Section 4, and it is not a feeding behaviour any animal in this review performs, so the proposal, its dose and safety limits, and the controlled human trial evidence supporting it are set out there.

#### 6.1.5 Geophagia-inspired detoxification and gut health management

Purified montmorillonite/kaolinite could adsorb bacterial toxins and reduce inflammation. Clay nanoparticles may serve as delivery vehicles for polyphenols and prebiotics. Clay binders are already in veterinary and feed-additive use for mycotoxin adsorption, but purified clay therapeutics are E2 to E3 for related human indications and have not been tested against a foodborne toxin exposure (E4 for that use). Binding reduces how much toxin is present, which Section 1.1 treats as the chemical-hazard equivalent of resistance rather than as immune enhancement; dosing, micronutrient binding and long-term microbiome effects are the open safety questions.

#### 6.1.6 Integration with traditional and ethnoveterinary knowledge

Ethnoveterinary plants such as Maesa lanceolata and Bolusanthus speciosus, introduced in the pillar above, are the leads considered here (149, 150). Standardisation of the extract, safety profiling and controlled efficacy testing in humans are the steps that would move them past *in vitro* screening, which is where the evidence for them stops at present (E4).

## 7 Extreme digestive chemistry and specialized immune mechanisms and application

Animals have evolved strong defences that create hostile environments for pathogens. These defences offer insights for preventing foodborne diseases in humans. One important defence is gastric hyperacidity (7, 71). Species like vultures and wandering albatrosses (Tier A) maintain highly acidic stomachs (pH ~1), sharply reducing the viable bacterial load of ingested food (7, 71). These species also carry distinctive gut communities that co-occur with tolerance of pathogens such as *Bacillus anthracis* and *Salmonella*. Likewise, formicine ants acidify their crops using formic acid venom, which sharply reduces survival of ingested pathogens while relatively sparing acid-tolerant symbionts (3).

Moreover, several lineages regulate inflammation at the gut surface while keeping antimicrobial capacity intact. Teleost fish possess abundant gut γδ T cells that secrete IL-10 and TGF-β, regulating immunity and preventing inflammation (151). Mice have Paneth cells that secrete defensins activated by matrix metallopeptidase 7 (MMP-7), maintaining gut homeostasis (152). The production of antimicrobial peptides (AMPs) in animals like bats (cathelicidins) and primates (theta-defensins) provides rapid, broad-spectrum antimicrobial action in the source species; Section 6.1 sets out why this does not translate into broad-spectrum systemic antibiotics for humans (153, 154). Antimicrobial peptides primarily engage innate immunity for rapid protection. Their ability to enhance adaptive responses is still under investigation and currently considered **emerging.**

Teleost fish release specialized immunoglobulins (sIgT) to safeguard the gut from pathogens, presenting a model for mucosal vaccines (30). sIgT and mucosal immunity mechanisms inform both innate barrier enhancement and adaptive mucosal vaccine design (e.g., oral vaccines eliciting robust secretory IgA).

### 7.1 Clinical translation of bio-inspired digestive chemistry and specialized immune mechanisms

Gastric acidity is a chemical barrier rather than an immune effector. Antimicrobial Peptide Therapeutics engage innate immunity directly while also supporting adaptive mucosal immunity. Controlled pre-exposure and nutritional immune modulation act on innate responses.

#### 7.1.1 Bio-inspired gastric acid modulation and microbiome engineering

- pH-Stable Delivery and Barrier Preservation: Scavenger hyperacidity is not a template for augmentation. Human gastric pH cannot be driven toward 1 without mucosal injury and impaired micronutrient absorption, and Section 8.2 sets out why we treat this adaptation as one the framework rejects. The defensible inference runs in the opposite direction. Gastric acid is a conserved barrier already doing substantial work, and its pharmacological removal is a modifiable risk, so the translational targets here are preservation of the existing barrier and pH-stable formulation of orally delivered agents, including probiotic coatings that survive stomach passage. Both are compatible with human physiology, and neither requires raising gastric acidity. The two differ in maturity and should be read separately. Barrier preservation rests on human observational evidence that suppressing acid raises the risk of enteric infection (E3), whereas pH-stable delivery to the gut lumen is a formulation problem that has not been solved for any foodborne indication (E4).

#### 7.1.2 Antimicrobial peptide therapeutics and mucosal immunity

Animal-derived immune mechanisms are considered here as adjuncts and niche agents rather than as replacements for conventional antibiotics, for the reasons set out in Section 6.1:

- Engineered AMP Therapeutics: Bat cathelicidins and primate theta-defensins could serve as templates for peptide development in the niches identified in Section 6.1 rather than as broad-spectrum systemic antibiotics (E4). Enhanced stability and specificity would have to be achieved through peptide engineering rather than assumed. While bat cathelicidins and teleost sIgT provide compelling molecular templates, the development of stable, orally deliverable AMP therapeutics or sIgT-inspired mucosal vaccines for foodborne pathogens is still at an **early preclinical stage**. Significant advances in peptide engineering, delivery systems, and immunogenicity profiling will be required before these can be considered viable clinical candidates.
- Mucosal Vaccine Platforms: Teleost fish’s specialized immunoglobulins (sIgT) and mucosal immunity mechanisms could inform the development of oral vaccines and antibody-based therapies for enhanced gastrointestinal protection against enteric pathogens. This is a mechanistic inspiration rather than a candidate intervention. The work is confined to fish, mammals use IgA in the equivalent mucosal role, and no oral vaccine or antibody product built on this template has been tested against a foodborne pathogen in a mammal (E5).

#### 7.1.3 Controlled pre-exposure and nutritional immune modulation

Nutritional immune modulators and controlled low-dose pre-exposure could prime innate responses while maintaining gut microbiome homeostasis. The formicine ant analogy is limited: what carries over is the principle of a brief, dosed exposure and not the acid itself. No such regimen has been tested against a foodborne indication in people (E4).

Note: Approaches in this pillar primarily target innate rapid responses and mucosal barriers, with potential to enhance adaptive memory (e.g., via better antigen presentation or trained immunity).

### 7.2 Antimicrobial peptides: a balanced assessment

Antimicrobial peptides recur throughout animal defence, and their breadth of activity has made them a durable candidate class. The translational record does not support presenting them as general replacements for conventional antibiotics, but it does identify settings in which the class already performs. We set out both.

Clinical programmes over three decades, including pexiganan, omiganan and iseganan, failed on systemic and broad indications; the agents of this class in use are predominantly topical, non-systemic, or lipopeptides with distinct pharmacology (155, 156). The obstacles are consistent: cationic amphipathic peptides discriminate imperfectly between microbial and mammalian membranes, giving a narrow therapeutic index, as the polymyxins show in a drug already in clinical use; peptides are protease-degraded, rapidly cleared, and lose activity at physiological ionic strength, so low-salt assays overstate potency; delivery is a particular obstacle here, since acting in the gut lumen implies oral administration through gastric acid and pancreatic proteases; and host defence peptides are not immunologically neutral, LL-37 being a T-cell autoantigen in psoriasis that forms immunostimulatory complexes with self-nucleic acids (157).

Resistance bears on the rationale for the class. Exposure to therapeutic antimicrobial peptides can select for resistance to endogenous human defence peptides, so treatment may degrade the patient’s own innate immunity; cross-resistance profiling should therefore be a gating experiment rather than a late consideration (158). More consequentially, colistin is a polymyxin, and its use in food animals is associated with plasmid-mediated mcr-1 resistance, now reported globally: resistance in this class can be horizontally transferable and mobile through the food chain (159). Any proposal to address antimicrobial resistance by deploying these peptides in the food system must account for this precedent, which is the principal constraint on the approach.

The realistic applications are narrower than often claimed but, for foodborne disease, closer to hand. Food processing and preservation is where the class already functions at scale, nisin being an established food-grade antimicrobial active against Gram-positive pathogens including Listeria (E1) (160). Engineered analogues using D-amino acids, cyclisation or peptidomimetic backbones improve stability in preclinical testing and can widen the therapeutic index in those systems, though they do not address resistance (E4). Host defence peptides acting as immunomodulators rather than microbicides engage host signalling and impose little direct selection on the pathogen; in our assessment this is the most promising direction in the pillar and warrants dedicated investigation. Pre-harvest feed applications are retained only on the condition, from the mcr-1 experience, that agents used in food animals must not share a resistance mechanism with a clinically important human drug.

## 8 Integrated host-microbiome defence: gut microbiome dynamics and host coevolution in animals

Animals have also developed integrated host-microbiome systems that enhance resistance to pathogens (161). These systems, which include pathogen exclusion, immune modulation, and epigenetic regulation, provide a strong defence against foodborne threats (162, 163).

The animal gut microbiome combats pathogens through competition for nutrients and direct attacks. For example, *Fusobacterium* and *Bifidobacterium* are associated with reduced abundance of harmful microbes, while *Janthinobacterium lividum* produces violacein, which antagonises fungal pathogens (162). *Bacteroides fragilis* employs the Type VI secretion system (T6SS) to kill competitors, including other gut *Bacteroidales* (74). Short-chain fatty acids (SCFAs) generated by fermentative bacteria like *Clostridiales* and *Ruminococcus* inhibit pathogen growth, lower gut pH, and strengthen gut barrier integrity (164, 165). In Tibetan pigs, *Lactobacillus* and *Bifidobacterium* are associated with pathogen exclusion through nutrient depletion and producing bacteriocins (166).

These microbiome dynamics are the result of deep host-microbe coevolution shaped by dietary pressures (167). The extensive use of agrochemicals has also pushed the development of advanced resistance strategies. For instance, the bean bug’s specialized midgut organ houses *Burkholderia* bacteria, which break down pesticides and integrate microbial detoxification into the host’s defence (Tier B) (168). The parasitoid wasp *Nasonia vitripennis* is cited for mechanism only (Tier B); exposure to the herbicide atrazine alters its gut microbiome to favour Serratia marcescens, an association reported alongside atrazine degradation and resistance in the following generation (M1) (169). This points to the possibility of building beneficial microbiomes that neutralise foodborne toxins, outcompete pathogens, and modulate LPS to prevent inflammation, enhancing food safety (72, 170, 171).

Microbial metabolites like SCFAs can influence host immunity by regulating epigenetic processes. Butyrate, a histone deacetylase inhibitor, supports the differentiation of regulatory T cells (Tregs), which are essential for immune tolerance (163). DNA methylation at immune gene promoters (e.g., Tlr4) also regulates immune responses and prevents harmful inflammation (172). An exciting emerging frontier is cross-kingdom RNA signalling. Host-derived microRNAs (e.g., miR-146a, miR-155, miR-21) have been reported to be taken up by gut bacteria and to alter bacterial gene expression, including virulence factors and quorum-sensing circuits in pathogens such as Salmonella and Pseudomonas. Conversely, certain bacterial small RNAs are reported to influence host immune gene expression; the evidence in both directions is associative and the claim is graded M1 in Section 7.1 (173). Plant-derived miRNAs from the diet can survive gastrointestinal transit and exert functional effects on bacterial growth or host inflammatory pathways (174). While the full therapeutic exploitation of these mechanisms is still in early stages, the existence of bidirectional miRNA-mediated communication provides a rationale for developing miRNA mimics, antagomirs, or engineered microbial strains that deliver specific small RNAs to fine-tune both microbiome composition and host immune tone against foodborne pathogens. Primary targets: gut epithelial cells, lamina propria immune cells, and microbiome consortia. Cross-kingdom miRNA signalling and engineered microbial strains remain speculative for clinical use. Significant validation in humanized models is required.

This integrated host-microbiome system allows for microbial immune priming. Invertebrates like the Pacific oyster can achieve lifelong immunity against pathogens such as Ostreid *herpesvirus-1* through microbial exposure early in life (175). That route is microbiome-mediated and is distinct from the host epigenetic transfer reported for the same species in Section 12; the two should not be read as one mechanism. In dairy cows, gut microbiome composition is associated with the functions of peripheral immune cells (M1) (176). Similarly, mice given bat gut microbiota show tolerance of influenza virus infection with reduced immunopathology, an effect associated with microbiota-derived isoflavones including daidzein and genistein; this is a heterologous transfer into mice rather than an observation in bats (M3) (177).

### 8.1 Strength of causal evidence for microbiome claims

Claims in this section are classified on the five-level causal scheme defined in Section 1.3, because causal strength varies widely across the microbiome literature (50). Several rest on compositional association alone (M1): Tibetan pig Lactobacillus and *Bifidobacterium* exclusion, the dairy cow immune-cell association, the *Nasonia* and Serratia marcescens case, and the observation that *Fusobacterium* and *Bifidobacterium* are found alongside reduced abundance of harmful microbes. Cross-kingdom RNA signalling is likewise M1, and the underlying mechanism remains contested.

Others rest on substantially stronger evidence. *Bacteroides fragilis* Type VI secretion antagonism is supported by defined effector and immunity proteins and demonstrated antagonism of other gut *Bacteroidales* (M4) (178). *Burkholderia*-mediated detoxification in the bean bug rests on a symbiont transfer experiment showing that acquisition confers resistance (M3 to M4) (179). Butyrate-driven regulatory T cell induction rests on germ-free experiments in which the isolated metabolite reconstitutes the phenotype (M4), among the strongest causal chains in this review (180). Transfer of bat gut microbiota conferring tolerance to influenza virus in mice is a heterologous transfer experiment (M3) (177). Faecal microbiota transplantation and defined live biotherapeutic products for recurrent *Clostridioides difficile* infection are supported by randomized trials and two approved products (M5) (181, 182).

One caveat applies at M3. Transfer into gnotobiotic rodents demonstrates sufficiency in that model, not causation in humans; donor and recipient differ in composition, colonisation behaviour and immune context, and the interpretive limits of human microbiota-associated rodent models are well documented and apply to every M3-level claim here (51, 180).

### 8.2 Community-level function and the limits of single-target translation

Much of what the gut microbiota does for its host is a property of the community rather than of any member of it. Colonisation resistance is the clearest case. Pathogens are held down by competition for nutrients and for adhesion sites, by bile acid transformation, by short-chain fatty acid production and by the physical state of the mucus layer, and these activities are spread across many taxa rather than concentrated in one (183). The host is part of this system rather than its audience. The bile acids the microbes transform are secreted by the host, the mucus layer and the adhesion sites being competed for are host tissue, and short-chain fatty acids act on host receptors, so the function belongs to the host and its microbes together rather than to the microbial community alone. Two features of such systems matter here. The first is functional redundancy: a function can survive the loss of any single member, which is why removing one organism often changes nothing, and why an organism that looks dispensable in a knockout may still be part of what produces the effect (184). The second is that members do not simply add up. What a defined consortium does is often not predictable from its members measured alone, because interactions among three or more species change what pairs of them do (185). A community function is therefore not a sum of single-organism functions waiting to be itemised.

This has a direct consequence for how the M ladder should be read, and it is not the obvious one. M4 asks whether one identified microbe, gene or molecule is responsible. For some functions that question has an answer, as Section 8.3 shows for butyrate (180). For others the question is malformed, and a failure to reach M4 then reflects the structure of the system rather than a deficiency in the evidence. A claim that stalls at M2 or M3 because no single member can be shown to be responsible is not thereby a weak claim; it may be a community-level claim tested against the wrong postulates. The ecological form of the argument applies in those cases (52), and we do not promote such exemplars to M4 by nominating a candidate organism on circumstantial grounds.

The point also changes what passes the decomposability gate of Section 1.4. A community function fails that gate when it is put forward as a single molecule or a single strain, and passes it when the translatable unit is redefined as the community itself. This is the gate as Section 1.4 states it, applied at the level at which the function operates, rather than a relaxation of the gate after a candidate has failed it. That redefinition is what the SPLIT verdict in **Figure 1** records, and it is not a lowering of ambition: the therapeutic unit becomes a consortium rather than a compound. The field has already made this move. Faecal transplantation transfers a whole community of unknown composition, defined live biotherapeutic products replace it with a specified set of organisms, and SER-109, a purified spore consortium, reached randomised trials and approval for recurrent *Clostridioides difficile* infection on that basis (181). The direction of travel is from an uncharacterised community toward a defined one, not from a community toward a molecule.

Four practical consequences follow for reading the microbiome findings in this review. Potency cannot be specified as an active ingredient, so release criteria and trial endpoints shift toward composition, engraftment and restored function, measured for instance as a bile acid or short-chain fatty acid profile, rather than toward the concentration of one substance. Risk shifts with them, because a product defined by its members carries whatever else travels with those members, which is the argument for defined consortia over donor material. And a negative trial of one organism does not refute a community-level hypothesis: the *Oxalobacter formigenes* case in Section 8.3 is exactly that, an organism carrying what looked like a single function which did not reproduce the effect when given alone. Finally, because the host is part of the system, the same consortium need not behave the same way in every recipient: what a community does depends on the community and the physiology it arrives into. Section 7.1 makes this point for transfer experiments, where donor and recipient differ in composition, colonisation behaviour and immune context, and Section 11 makes it for animals raised in an impoverished microbial environment. A trial of a consortium-scale product therefore has to treat the recipient’s starting state as a variable rather than as background. Where an exemplar here rests on a community-level function we report it at that level and rank it as a consortium-scale proposal, which is why defined consortia rather than isolated strains appear in the near-term group of the Summary.

### 8.3 Clinical translation of host-microbiome synergy for foodborne pathogen defence

Advanced Probiotic Consortia primarily enhance innate immunity through competitive exclusion and barrier reinforcement while supporting adaptive immunity via SCFA and Treg induction. Metabolite-Based Immunomodulation acts on both innate and adaptive immunity through Treg differentiation. Cross-Kingdom Signaling and Bile Acid Metabolism mainly influence innate and immunometabolic pathways with effects on adaptive tone.

#### 8.3.1 Advanced probiotic consortia and microbial ecosystem therapy

Agrochemical Bioremediation Probiotics: Building on animal models, whether targeted probiotic solutions can be developed for clinical and agricultural use is an open question, at the maturities marked below. Engineered consortia inspired by *Burkholderia* symbionts in the bean bug could be investigated as livestock feed supplements that degrade agrochemicals such as atrazine and organophosphates in the gut (E5; demonstrated in insects, untested in livestock), with prevention of toxic residue transfer into the human food chain as the intended endpoint. This represents an emerging application that extends established probiotic concepts into agricultural and food-chain safety. While the underlying microbial degradation mechanisms are well-supported in animal models, translation to safe, effective, and regulatory-approved consortia for livestock or human use is still in the developmental phase and will require rigorous strain selection and safety validation.

- Pathogen-Competitive Designer Communities: Inspired by defensive microbial alliances, whether multi-strain probiotics can be engineered to outcompete pathogens via nutrient blocking, bacteriocin production and Type VI secretion systems is untested for a foodborne indication (E4). The clinical validity of this approach is demonstrated by faecal transplantation and the defined products described in Section 7.2 for recurrent *Clostridioides difficile* infection (181, 182), and by Staphylococcus hominis excluding *S. aureus* in atopic dermatitis (186).
- Antifungal biocontrol agents: based on models like violacein-producing *Janthinobacterium lividum*, whether microbial additives could be developed to target fungal contaminants in food systems is untested (E4), and reduced mycotoxin exposure would be the endpoint to demonstrate.

#### 8.3.2 Metabolite-based immunomodulation

Butyrate-based interventions represent an emerging strategy rather than a tested one. Butyrate-driven regulatory T cell induction is well supported as a mechanism, the isolated metabolite reconstituting the phenotype in germ-free animals (M4), but it has not been tested against a foodborne endpoint in people (E4). While the preclinical effects are strong, clinical evidence for preventing foodborne infections in humans is still limited. Microbial metabolites activating aryl hydrocarbon receptor (AHR) pathways could be harnessed for dual toxin clearance and immune regulation, subject to the limit set out in Section 4: intermittent, substrate-aware exposure only, since sustained agonism is not defensible (122, 127, 128). Histone acetylation and DNA methylation changes of this kind are in principle reversible, although Section 12 notes that a state built to persist is not easily undone, and can be induced in humans through high-fibre and polyphenol-rich diets, defined microbial consortia, or pharmacological epigenetic modifiers. The primary targets are circulating and tissue-resident monocytes and macrophages, dendritic cells, and mucosal T cells. Measurable outcomes are increased regulatory T cell and IL-10 levels and reduced excessive cytokine release, both tolerance-directed in the terms of Section 1.1; improved clearance on secondary challenge, which is resistance-directed; and shifts in chromatin accessibility or histone marks assayed by ATAC-seq or ChIP in peripheral blood mononuclear cells. Mechanistic support from animal and human cell studies is strong, but controlled human trials for foodborne indications have not been done.

#### 8.3.3 Cross-kingdom signalling and immune priming

Whether host-derived microRNAs that fine-tune bacterial growth could be developed into agents that modulate microbiome composition is an open question (E5). Whether early-life interventions inspired by Pacific oyster lifelong immunity could confer lasting protection against foodborne pathogens is unknown (E5); Section 12 sets out why the oyster is kept as a contrast case rather than as evidence that a primed state can be transferred in mammals. Host-microbiome cross-kingdom RNA signalling and early-life microbial priming strategies inspired by invertebrates such as the Pacific oyster are mechanistically fascinating but remain speculative for clinical application at present. Substantial fundamental research and validation in humanized models are needed before these concepts can inform practical interventions against foodborne pathogens.

#### 8.3.4 Bile acid metabolism and immunometabolic interventions

Whether microbial bile acid metabolites could be developed into agents enhancing enteric resistance is untested (E4). Microbiota-derived isoflavones including daidzein and genistein are associated with tolerance of influenza infection in mice given bat gut microbiota (M3); they have not been tested as adjunctive therapies against any foodborne pathogen (E4). Modulating bile acid metabolism or harnessing specific microbial metabolites such as those observed in bats offers an interesting emerging avenue for enhancing enteric resilience. However, the complexity of bile acid signalling and the need for precise, context-dependent modulation mean that these approaches are still largely at the preclinical stage and require careful mechanistic and safety studies before clinical translation.

By translating these animal-microbe partnerships into advanced probiotics, metabolite therapies, and immune-priming strategies, the aim is to move from reactive to proactive management of foodborne disease.

Overall for microbiome pillar: Advanced probiotics, postbiotics (butyrate), and competitive exclusion consortia are prioritised by the present framework for the near to medium term, on the grounds that agents and routes already exist, risk is acceptable and implementation complexity is lower. This is a judgement internal to the framework rather than a health-economic finding, and no formal cost-effectiveness analysis is presented here. Engineered or miRNA-based approaches are longer-term and more speculative.

## 9 Constraints, negative cases and the limits of biomimicry

### 9.1 Six obstacles to transferring a trait between species

Six kinds of obstacle stand between an animal adaptation and a human treatment, and the mismatch works in both directions: a trait shaped by life as a bat or a vulture cannot be assumed to behave the same way in a person. The first is ancestry: humans may simply not carry the gene or pathway involved. The second is context. A trait can depend on other changes that evolved alongside it, as the loss of the PYHIN genes in bats depends on their particular interferon settings, their flight-related protein handling and their unusual lifespan; the same loss in a genome without those companions is not the same trait. The third is body plan and physiology: size, metabolic rate, how fast food moves through the gut, and lifespan. Rumen microorganisms degrade trichothecenes, aflatoxins and several plant toxins (187), but that capacity belongs to an anaerobic chamber held upstream of the acid stomach and cannot be moved to a human without moving the organ (**Supplementary Table S1**). The fourth is life history. Immune investment is tuned to an animal’s breeding schedule rather than to a long human health span, so tolerating a lasting infection can be a good evolutionary bargain and a poor clinical one. The fifth is that the same gene usually does other jobs in humans: AIM2 and IFI16 detect DNA loose in the cell, and NRF2 is involved in tumour drug resistance. The sixth is a difference in what is being optimised: animal adaptations serve one individual’s reproductive success, whereas public health serves populations.

### 9.2 Adaptations that do not translate

A framework that cannot reject is not a framework. Four cases are excluded by our criteria; in three, exclusion redirects rather than ends the enquiry. These four are developed in full because each one changes what the review recommends. Other adaptations were removed earlier, either because the hazard falls outside the scope set in Section 1.2 or because a gate in Section 1.4 excluded them before ranking. **Supplementary Table S1** lists representative cases of both kinds with the stage at which each was removed and the reason.

The vulture and albatross stomach reverses its lesson when applied to people. Fasting gastric contents in people already fall to about pH 1 to 2, so the distance between a human and a scavenger stomach is smaller than it first appears. Luminal pH is also not the pH the mucosa itself experiences, since the adherent mucus and bicarbonate layer holds the epithelial surface much closer to neutral, and the two should not be equated. The case against augmentation therefore does not rest on an inability to reach a low pH. It rests on three points: humans already carry a strong acid barrier; raising acidity beyond the normal physiological range has not been shown to give clinical benefit; and deliberately increasing acid exposure carries its own gastrointestinal risks, including mucosal injury and reflux. What the tolerability gate rejects is sustained acidity above the normal human range, not the transient low pH that already occurs. The useful inference runs the other way. Stomach acid is an ancient barrier already doing real work, and removing it with medicines is a risk we can control: long-term use of acid-blocking drugs is associated with more community-acquired gut infections (188). Comparing many mammals and birds shows that stomach acidity rises with how much carrion and raw meat a species eats, and human acidity is already high for our place in that range (189). The lesson is to protect the barrier we have, not to strengthen it further.

The sea urchin’s enormous set of immune receptors is a substitute, not an upgrade. Sea urchins have no adaptive immune system, so they inherit a vast range of receptors to cover threats that vertebrates handle by building new receptors during life. Adding a comparable set to an animal that already has adaptive immunity would be expected to make the immune system attack the body itself. The adaptation is still valuable outside the body, as a design for detectors that recognise many targets at once, where breadth costs nothing. The horseshoe crab supplies the precedent: its clotting cascade became a licensed test used worldwide. On that basis this is one of the more workable ideas in this review, provided it is built as a diagnostic device rather than a therapy.

The bats’ loss of the PYHIN genes sits inside a set of other changes humans do not have, and in humans AIM2 and IFI16 still do necessary work. What could be copied is the resulting state, inflammation held in check, rather than the missing genes themselves, and that state can be approached temporarily with drugs that damp the NLRP3 inflammasome, several of which are in clinical development (E2) (190). Deleting a gene and reproducing an outcome are different operations. The toothed-whale loss of the MX1 antiviral gene we keep as a case with no plausible human route at all, because a filter that finds value in everything is not filtering.

### 9.3 Can acting on one pathway reproduce a whole-body trait?

Can a complex, whole-body trait be reproduced by acting on a single pathway? It depends on whether the trait can be taken apart, and that is testable. Diets rich in carotenoids were linked to less lung cancer, yet giving smokers purified beta-carotene increased it: reducing a whole food to one molecule did not preserve the benefit, it reversed it (191). Blocking MD2-TLR4 with eritoran failed to reduce deaths in severe sepsis despite a convincing mechanism on paper (192, 193). Both bear on proposals made here. The successes share the opposite feature. Faecal transplants and defined live bacterial products move a whole community to fix a problem that belongs to a community, as Section 7.2 sets out, and BCG sets off a whole programme rather than supplying one missing molecule. A mechanism can be taken apart when one isolated substance reproduces the entire effect, as butyrate does when it induces regulatory T cells in the colon of germ-free animals, and those mechanisms suit single-drug treatment. A third case sits between these two. *Oxalobacter formigenes* degrades dietary oxalate and looks like one organism carrying one function, which is the profile that should transfer well. A meta-analysis of five randomised trials in 208 patients with primary hyperoxaluria, in whom the oxalate load is endogenous rather than dietary, found no significant effect on plasma or urinary oxalate (194). Replacing a function that a community performs is not the same operation as replacing a molecule, even when a single species can be named (**Supplementary Table S1**). Where only the whole thing works, or where the partner changes are missing in the recipient, treatment has to be aimed at the level the mechanism actually operates on. The proposals we rank highest for the near term are the system-level ones, which is what this test predicts.

### 9.4 Adaptations are trade-offs, not improvements

Every adaptation described here was paid for. Immunity is expensive and competes with growth and reproduction, so animals invest what is worthwhile rather than the maximum, and the results are narrow. Vultures eat carrion that would kill most vertebrates, yet Gyps vulture populations across the Indian subcontinent collapsed because cattle carcasses carried residues of the drug diclofenac, which causes kidney failure in these birds at tiny doses. The very specialisation that gave them their advantage delivered the poison, and several species are now critically endangered (195, 196). Other costs are comparable. Bat tolerance means staying infected and shedding virus; the sea urchin’s receptor set is expensive to carry; a strongly acid stomach narrows the microbes that can live there; the panda’s detoxification cannot be separated from its extreme diet; and expanded detoxification genes cost energy, with the resistant versions often reducing fitness once the chemical is gone, although by how much is debated (56, 197). We therefore avoid saying that animals defend themselves better than humans, a claim that means little without stating in what respect, measured how, in which environment and at what price. Human defences are largely outside the body: cooking, fermenting, preserving, refrigeration, sanitation and vaccination, which measured by lives saved from foodborne illness are extremely effective. Our vulnerability comes from the scale and structure of modern food systems and from an immune system calibrated by a different environment, not from missing equipment, and the case for copying animals rests precisely on that equipment being intact and under-used.

## 10 Convergent evolution: the same defence arising in unrelated animals

When the same solution evolves more than once in unrelated animals, it is a general answer to a recurring problem rather than an accident of one family’s history. This differs from a solution shared by inheritance. A mechanism humans inherited from a distant common ancestor is probably still present in us and can be switched on; one that arose independently shows the design is reachable, and where humans lack it, it would have to be engineered. Acidifying the compartment where food is held evolved separately in Old World and New World vultures, which are not close relatives, again in albatrosses and petrels, and by different chemistry in formicine ants (3, 7, 198); the comparative gradient of stomach acidity with carrion and raw-meat feeding is set out in Section 8.2. Avoiding contaminated food, eating clay, and recruiting detoxifying bacteria have likewise arisen independently in several groups. By contrast, PYHIN loss in bats, receptor expansion in sea urchins and antiviral gene loss in whales each happened once.

How this classification lines up with our translatability assessment is the most suggestive internal check we can offer for the selection rule this review proposes. By the standard set out in the Summary it is not validation, because the agreement is measured on the material the rule was built from. Mechanisms with several independent origins fall into the near-term group; those with a single origin fall into the longer-term group or are excluded. Convergence, whether a mechanism can be taken apart, and the strength of the causal evidence were judged separately, yet they rank the mechanisms in the same order. That agreement is consistent with translational promise being a real property of a mechanism rather than a reflection of which examples we happened to choose, though only an independent case set could establish it: an adaptation that has evolved repeatedly, works through an identifiable single substance, and rests on solid causal evidence is the better candidate.

## 11 Evolutionary consequences of biomimetic intervention

Copying animal defences is often argued to relieve the pressure that drives antibiotic resistance, but any treatment that changes how well a pathogen survives applies pressure of its own. Treatments differ in how strong that pressure is, what it acts on, and whether bacteria escape by mutating or by picking up a gene from a neighbour. Bacteria can become resistant even to interference with their chemical signalling, so anti-virulence approaches are not immune to evolution either (199, 200). Several proposals here have known escape routes. Antimicrobial peptides act on the bacterial membrane, and the mcr-1 precedent set out in Section 6.1 shows that resistance in this class can be mobile. Acidifying the gut selects for acid-tolerant bacteria, and acid-adapted Escherichia coli O157:H7 already survives better in acidic foods (201). Bacterial mixtures that crowd out pathogens select for pathogens that find another way to feed, as Salmonella does by using compounds released during inflammation (202). Engineered live organisms raise a different worry: not resistance, but the difficulty of taking them back once released.

Treatments that copy tolerance differ in kind. They put no pressure on a drug target, which is their attraction, but theory predicts the opposite effect at population level: reducing pathogen numbers lowers how common an infection is, whereas tolerance leaves people infected and still passing the organism on, which can make it more common (39). A treatment that makes an individual less ill while leaving them carrying the organism may therefore raise the total burden, which for foodborne disease limits where such treatments are appropriate. We therefore propose that these interventions be introduced with a plan for managing resistance: monitoring susceptibility in the target organism and in bystanders, combining or rotating agents so that escape requires more than one change, keeping untreated populations as a refuge, and never using in food animals an agent that shares a resistance mechanism with a drug that matters in human medicine.

### 11.1 Long-term safety of interventions that are meant to last

The consequences discussed so far fall on the pathogen. Some of the proposals ranked here also leave a lasting mark on the person who receives them, and that deserves separate treatment. A conventional drug is cleared, and stopping it ends the exposure. A trained myeloid compartment, an engrafted microbial community and a re-set inflammatory threshold are not like that. Their durability is the attraction, because one administration buys a long effect, and it is also the hazard, because the same property makes them hard to withdraw when the effect turns out to be unwanted. Two questions therefore belong to every durable proposal and not only to the ones that look risky: what happens if the change runs in the wrong direction for this person, and what can be done about it afterwards.

Persistent immune training is the clearest case. As Section 12 sets out, protection outlasting the circulating monocyte has to be written into bone marrow progenitors (203), and once it is written no agent reverses it. That the same mechanism can work against the host is not speculative. In mice a western diet induces NLRP3-dependent reprogramming of myeloid progenitors that persists after the diet is corrected (204), and in people oxidised LDL trains monocytes into an inflammatory state implicated in atherosclerosis (205). The opposite manoeuvre carries its own cost. Damping the inflammasome, which is the route this review retains from bat biology (190), lowers cardiovascular events in trials of interleukin-1 beta blockade, but fatal infection and sepsis were more common than on placebo (206). Raising innate reactivity and lowering it are both two-sided, and a foodborne prevention strategy would be given largely to healthy people, for whom the acceptable level of that risk is lower than for someone being treated for established disease.

Microbial interventions raise the problem in another form, because a live product replicates. Stopping it does not remove it, and an organism that engrafts has in effect been given permanently. Harm can also arrive with the product rather than from it: faecal transplantation has transmitted an extended-spectrum beta-lactamase-producing *Escherichia coli*, causing bacteraemia in two recipients and one death, after which trials were halted while donor screening was revised (207). Within a consortium, resistance determinants can move between members by horizontal transfer, the same route that carried mcr-1. And as Section 7.2 sets out, a product whose activity is a property of the community cannot be specified by an active ingredient, so an unexpected effect is correspondingly harder to attribute and harder to undo. Loss of diversity and displacement of resident taxa belong in the same account, because an intervention judged on one endpoint can move the community in ways that only show up on another.

Four rules follow, and they are why Section 1.4 ranks on risk and reversibility together rather than on risk alone. Where two approaches reach the same endpoint, the one whose effect decays is preferred, and a repeated intervention with a short memory is safer than a single permanent one. Any durable proposal should carry a stated exit: an agent that clears a live product, or a defined interval beyond which training is not re-dosed. People in whom a raised inflammatory set point is already harmful should be excluded rather than assumed to behave like the trial population, the point Section 12 makes for trained immunity. And monitoring has to run on the timescale of the intervention rather than of the trial: cardiovascular and autoimmune endpoints for immune training, colonisation and resistance-gene carriage for consortia, measured in years. Proposals that cannot meet these conditions sit in the long-term group of the Summary for that reason, and not only because the enabling technology is immature.

## 12 Evidence settings and their limits

Every setting in which animal defence can be studied removes something. Animals in the wild can be watched but not experimented on, so mechanisms are worked out instead in cell cultures, in captive animals, or in a different species used as a stand-in. Across the mechanisms reviewed here, the more precisely a mechanism is understood, the less natural the setting in which it was studied. Captivity adds to the problem: captive animals differ from wild ones of the same species in their gut bacteria, their diet and their immune function, and this reshaping has been documented in many mammals including giant pandas (208–210). Where a mechanism depends on gut bacteria, as the panda’s handling of cyanide largely does, evidence from captive animals may be describing an animal changed by captivity.

Laboratory animals have the opposite problem. Mice raised free of specific pathogens have immune systems that resemble a human newborn’s more than an adult’s, and letting them live in a normal microbial environment shifts them toward adult humans (211, 212). This matters directly for what we argue here. If human susceptibility comes from too little microbial exposure, then a germ-poor laboratory mouse reproduces the very deficit we want to correct, and will give the wrong answer about any treatment that works through microbial exposure, immune training or the gut barrier. We therefore recommend testing in animals kept in more natural microbial conditions, note that even closely related mammals do not always predict one another (213, 214) (a point on which the literature directly conflicts), and give most weight to mechanisms where field observation and controlled experiment point the same way.

## 13 Trained immunity: scope and limits

Trained immunity is a lasting change in how the body’s first-line immune cells behave, produced by chemical marks on their DNA and by shifts in their metabolism (215). It happens without the gene shuffling and cell selection that antibodies and T cells depend on, and the protection it gives is not tied to one particular germ, which is what separates it from adaptive immunity (57). How long it lasts is limited by how long the cells live: monocytes circulating in blood survive only days, so protection lasting months must be written into the bone marrow stem cells that produce them, which BCG has been shown to do (203). The effect fades, so in mammals trained immunity is neither lifelong nor passed to offspring, and it is not the same thing as immune priming in insects and shellfish, despite the shared vocabulary (216). In the Pacific oyster, parental exposure is followed by altered immune gene expression in offspring and reduced susceptibility to *Vibrio splendidus*, suggesting epigenetic mechanisms for immune transfer (217). That is transgenerational transfer of a primed state, which mammals do not do, and it is why the oyster is kept here as a contrast case rather than as evidence for trained immunity in humans.

Which direction it takes depends on the circumstances. Depending on which receptor is engaged, at what dose and for how long, human monocytes end up either trained or damped down, and beta-glucan can reverse the damped state caused by bacterial LPS (218, 219); training and suppression are two outputs of one system, the same dependence on dose and context set out in Section 4.1. Age, sex, previous exposures and metabolic health all shift the result. A lasting rise in immune reactivity is also not always desirable: oxidised LDL is the case set out in Section 10.1 (205). Section 10.1 sets out what follows for use at population scale. In the terms of Section 1.1, trained immunity mainly increases resistance. Two maturity labels apply here and should not be run together. BCG and beta-glucan are in approved human use for their licensed indications (E1), whereas trained immunity as a deliberate strategy against foodborne disease has not been tested in a controlled human trial and stands at E4. This section therefore describes a mechanism and its limits rather than putting forward an intervention ready for use; the graded proposals that draw on it are given in Section 3 and Section 7.

## 14 Summary and future research directions

We propose a shift of emphasis, moving from pathogen-centric to host-resilience-centric infectious disease management to reduce reliance on antimicrobials and broaden protective scope. As set out in Section 10, this shift relocates rather than removes selective pressure on pathogens, and interventions should be designed with that consequence in view. The Biomimetic Immunity Continuum provides a structured framework for this shift, organizing interventions from low-risk dietary and ecological mimicry to advanced molecular and cell-based therapies while explicitly prioritizing feasibility, reversibility, safety, and ecological responsibility. Three contributions follow from it. It supplies a vocabulary that separates avoidance, resistance and tolerance, which carry different therapeutic timing, different selection pressures and different population-level consequences. It supplies a selection rule that ranks candidates and, unusually for this literature, rejects some. And it yields a testable prediction, that among adaptations acting on conserved or reachable human pathways, translational value tracks conservation, accessibility, safety and deliverability rather than the magnitude of the animal phenotype; the three independent criteria assessed in Section 9 rank the mechanisms in the same order.

Four limitations bear on what we have argued. The choice of examples rests on our judgement rather than a systematic search, and Section 1.6 states plainly what that costs. Much of the mechanistic evidence comes from captive animals or from germ-free laboratory models, each of which removes part of the biology it is meant to represent (Section 11) and several proposals we retain, among them gut-targeted genome modulation, engineered exosomes, broad enzyme supplements and miRNA therapeutics, sit at E4 or E5, where the limiting step is an enabling technology rather than the underlying animal biology. A fourth limitation concerns the labels themselves. A candidate at E1 has reached use, not necessarily for this indication and not necessarily on strong comparative evidence, and a candidate at E5 may rest on a substantial experimental literature in the source species. The labels locate a proposal on the path to use, and should be read alongside rather than instead of the established instruments for evidence certainty and study design.

Reviewed against cost, regulatory complexity and time to implementation, the pillars do not rank equally. Dietary and phytochemical interventions, clay and mineral binders, defined probiotic and postbiotic consortia, and biosensor diagnostics are the near-term group: low cost per person, existing regulatory routes through foods, supplements or devices, and in several cases established products already on the market. Heat-shock inducers, epigenetic primers and engineered antimicrobial peptides form a middle group whose obstacles are formulation and stability rather than principle. Recombinant gut-luminal enzymes, gut-targeted genome modulation, exosome and miRNA therapies and synthetic receptor arrays are the longest-term and most expensive, and are best reserved for high-risk or outbreak settings rather than general introduction. This ordering follows the decision rule set out in Section 1.4 and applied in **Table 2**, and the evidence levels of Section 1.3, rather than the appeal of the underlying animal biology. The statements about cost and complexity in this paragraph are the framework’s own qualitative ratings, not the result of an economic evaluation.

Three research directions follow from the framework rather than from the field at large. The prediction set out in Section 1.4, that among adaptations acting on conserved or reachable human pathways, translational value tracks conservation, accessibility, safety and deliverability rather than the magnitude of the animal phenotype, can be tested against the next decade of animal-inspired candidates, and it can fail. The decomposability criterion in Section 8.3 should be applied prospectively rather than retrospectively: for each proposed mechanism, does an isolated mediator reproduce the effect, or does only the intact system do so? And where only the intact system does, can that system itself be specified and delivered as the therapeutic unit? And the mechanisms this framework ranks highest, defined microbial consortia and dietary interventions, are ready for controlled human trials with foodborne endpoints rather than surrogate markers.

### 14.1 How the framework could be validated, and how it could fail

Those directions concern the biology. The framework itself also needs testing, and because it is a decision rule rather than a mechanism it should be held to the standard that applies to one: it is useful only if it separates candidates that go on to progress from those that do not, and it earns nothing by being plausible. What the framework is built to predict should be stated plainly. It ranks candidates by how readily they can be developed, not by how well they will work. Progression and efficacy are different things: a candidate can move quickly into human testing and still fail to help anyone with a foodborne illness. Validation should therefore be scored against three endpoints kept apart rather than one. The first is progression, meaning entry into human testing for a foodborne indication. The second is efficacy, meaning benefit against that indication in a controlled trial. The third is implementation, meaning approval and routine use. We expect the rule to do best on the first, less well on the second and least well on the third, since efficacy turns on the biology of the intervention and implementation on cost, regulation and practice. A rule that picked developable candidates but not effective ones would be a partial success and should be reported as one. The first test is retrospective calibration. Assemble a set of animal-derived candidates whose fate is already known, score each on the three gates and the four ranking criteria, and ask whether the resulting order separates the arrivals from the failures. Cases are not scarce. On one side sit the horseshoe crab clotting cascade, which became a licensed endotoxin assay; geophagia-inspired clay binders, in veterinary and feed-additive use and at E2 to E3 for related human indications; faecal transplantation and the defined spore consortium that followed it; broccoli-sprout chemoprevention, which reached randomised trials; and BCG. On the other sit *Oxalobacter formigenes*, which passes every gate and then failed five randomised trials; the MD2-TLR4 antagonist that failed in severe sepsis despite a convincing mechanism; and purified beta-carotene, which reversed the benefit of the whole food. Two conditions turn this from an illustration into a test. Scoring has to be blind to the outcome, which means using the literature as it stood when the candidate was first proposed. And the case set has to be independent of the examples used here, because a rule cannot be validated on the material from which it was built.

Calibration on its own is weak, since almost any sensible rule beats chance. The sharper question is whether this rule beats the one the field uses implicitly, which is to rank candidates by how striking the animal phenotype is. Section 1.4 predicts that it does, and predicts further that within the set of candidates that pass the gates, phenotype magnitude adds little once conservation, accessibility, safety and deliverability are known. Scoring the same set both ways and comparing the two orderings against what actually happened is a direct test of that prediction, and it is the test we would most like to see run. A second comparison is worth including in the same exercise: ordering on the limiting step against ordering on a simple average of the four criteria. If the two agree on an independent case set, the distinctive machinery of this framework earns nothing and should be dropped.

The remaining tests are prospective. The groups in **Table 2** are predictions with dates attached and can be registered as such: we expect at least one near-term candidate, most plausibly a defined consortium or a dietary intervention, to enter a controlled trial with a foodborne endpoint within a decade, and expect none of the long-term group to do so on that timescale. That prediction concerns progression alone. Whether such a trial reports benefit is the efficacy endpoint, and whether an intervention enters routine use is the implementation endpoint; both should be recorded separately as they mature, and neither is claimed here. A second prospective test applies the rule to animal adaptations described after this review rather than to candidates already in it. As each new adaptation is reported it can be scored on the three gates and the four criteria, assigned a group before its fate is known, and that assignment lodged as a dated prediction. This is the cleaner of the two prospective tests, because the case set is independent of the material the rule was built from and no reconstruction of what was known at the time is required. A separate test concerns reproducibility rather than accuracy. Section 1.4 claims that the matrix makes the reasoning reproducible, not the result, and that claim is checkable by having two groups score the same candidates independently and reporting agreement cell by cell; disagreement concentrated on one gate would show that gate to be underspecified, which is more useful than disagreement spread evenly. Three outcomes would count against the framework as a whole: a candidate excluded by a gate reaching approved human use for the indication it was excluded from, ordering by phenotype magnitude outperforming ordering by conservation and accessibility, and the limiting-step rule proving indistinguishable from an average, and the ordering predicting progression while showing no relation to efficacy once trials report. We would rather see any of these established than see the framework adopted without being tested.

The contribution of this synthesis, if it has one, is not the catalogue of animal adaptations but the rule for choosing among them: a rule that others can apply, disagree with case by case, and show to be wrong.

## Supplementary material

Supplementary Table 1 lists representative candidate adaptations that were considered and excluded. The table covers examples removed at the scope stage (Section 1.2), at one of the three gates (Section 1.4), and during ranking on evidence or safety, and states where an exclusion redirects the enquiry rather than ending it. The table and the references cited in it are placed at the end of this manuscript, after **Table 1**.

Supplementary Table 2 sets out, level by level, what the E and M scales assert and what they do not

Supplementary Table 3 places both scales beside the established evidence frameworks.
