# Supplementary material for: Understanding and translating evolutionary adaptations in animals exposed to contaminated diets to guide strategies against human foodborne disease: a prioritisation framework
Source: Front Immunol. 2026 Oct 19;17:1883529. doi: 10.3389/fimmu.2026.1883529 (PMC13725789; doi:10.3389/fimmu.2026.1883529)
Supplement: Supplementary file 1 [file SupplementaryFile1.docx]

**Supplementary Material**

**Supplementary Table S1.** Representative candidate adaptations considered for this review and excluded, with the screening stage at which each was removed and the reason. The framework is applied in three successive steps. Scope comes first: an example that meets none of the three inclusion tiers defined in Section 1.2 is removed before any translational judgement is made. The three gates of Section 1.4 come next, and failing one removes a candidate from the ranking altogether rather than lowering its position. Candidates that pass the gates are then ordered on evidence, accessibility, risk and reversibility, and scalability, and a candidate can still be set aside at that stage on evidence or on safety. The examples below are representative rather than exhaustive, and are chosen to show each kind of exclusion at work. Where an exclusion redirects the enquiry toward a different and defensible target, that is stated. The four cases developed at length in Section 8.2 are not repeated here.

| **Adaptation and source animal** | **Hazard in the source species** | **Stage at which it was excluded** | **Reason for exclusion** | **What follows from the exclusion** | **Ref.** |
| --- | --- | --- | --- | --- | --- |
| Softshell clam (*Mya arenaria*): pore mutation in the voltage-gated sodium channel giving roughly 1,000-fold lower saxitoxin affinity | Paralytic shellfish toxin in ingested dinoflagellates; a Tier A hazard for humans | Gate 2: physiological tolerability | Humans carry the same channel family, so conservation is not the obstacle. The trait depends on altering the toxin-binding site of a channel essential to nerve and muscle function, and no tolerable route exists to reproduce that in a person. The adaptation also lets the clam accumulate more toxin, which raises rather than lowers human risk. | Excluded from the ranking. Retained as an argument for shellfish monitoring, which is surveillance rather than intervention. | (53) S1 |
| Monarch butterfly (*Danaus plexippus*): three substitutions in the alpha-subunit of the Na+/K+-ATPase | Cardenolides in milkweed; a natural plant poison under the WHO definition used here | Gate 2: physiological tolerability | The human orthologue is the digoxin receptor and is essential to cardiac and renal function. The substitutions were tolerated when introduced into *Drosophila* by genome editing, but that does not transfer: altering this pump in a human is not a survivable intervention. | Excluded. No redirect; the target itself is the problem. | (222) S2 |
| Mouse hepatic glutathione S-transferase A3, which conjugates the aflatoxin B1-8,9-exo-epoxide | Aflatoxin B1 in mouldy feed; a Tier A hazard | Gate 1: conservation | Mouse resistance depends on constitutive mGSTA3, whose catalytic efficiency toward the epoxide is orders of magnitude above any human alpha-class GST; human and non-human primate enzymes show no measurable activity toward it. There is no human counterpart to induce. | Excluded as a gene-level target, and the exclusion redirects: the upstream inducible route is the one this review ranks (broccoli-sprout and sulforaphane chemoprevention, Section 5). | (132, 133) S3,S4 |
| Rumen microbial de-epoxidation of trichothecenes and degradation of aflatoxins, ochratoxins, fumonisins and several plant toxins | Mycotoxins and plant toxins in forage; a Tier A hazard class | Gate 3: decomposability | The activity belongs to an anaerobic foregut community held upstream of the acid stomach. Humans have no equivalent compartment, and the capacity cannot be separated from the organ that houses it. | Excluded as a whole-system transfer. Isolated degrading organisms remain in use as animal feed additives, which is a food-chain route rather than a clinical one. | (187) S5 |
| *Oxalobacter formigenes*: obligate degradation of dietary and endogenous oxalate | Oxalate, a natural plant compound; a Tier A hazard | Ranking, on evidence | This candidate passes all three gates and entered the ranking. A meta-analysis of five randomised trials in 208 patients found no significant effect on plasma oxalate (p = 0.06) or urinary oxalate (p = 0.86), with no safety signal. | Excluded on evidence rather than on principle. It is the cautionary case for replacing a community function with a single organism, and is read here in the terms of Section 8.3. | (223) S6 |
| Pangolin (*Manis* spp.): pseudogenisation of IFIH1/MDA5 and ZBP1 | Viral infection; not an ingested hazard | Tier, then Gate 1: conservation and context | The same class of case as the bat PYHIN loss already analysed in Section 8.2. Both sensors are intact and doing necessary work in humans, and the trait depends on the rest of the pangolin sensor repertoire rather than on the deletion alone. | Excluded as redundant with a case already analysed in full. It adds no human node the review does not already consider. | (224) S7 |
| Tardigrade Dsup: a nucleosome-binding protein that reduces X-ray-induced DNA breaks when expressed in human cultured cells | Desiccation and radiation; not a foodborne hazard | Tier (scope), then Gate 1: conservation | The hazard falls outside the WHO foodborne definition adopted in Section 1.2. Independently, no human orthologue exists, so the protein would have to be introduced as a transgene rather than switched on. | Excluded. Noted only as evidence that a foreign protein can be made to act on a human nuclear target. | (225) S8 |
| Honey badger, mongooses, hedgehogs and pigs: convergent nicotinic acetylcholine receptor substitutions that block alpha-neurotoxin binding | Snake envenomation | Tier (scope) | Envenomation is not an ingested hazard under the definition used here, so the example meets none of Tiers A, B or C. It never reached a gate. | Excluded at the scope stage. Listed because it is among the most frequently cited animal resistance traits and a reader may expect to find it here. | (226) S9 |
| Grasshopper mouse (*Onychomys torridus*): Nav1.8 variant in which bark-scorpion toxin blocks the channel instead of opening it | Envenomation | Tier (scope) | Not an ingested hazard. The translational value is in analgesia, which is a different clinical problem from the one this review addresses. | Excluded here. A clear case where an animal is highly informative for a field outside this review, which is why scope is applied before merit. | (227) S10 |
| Komodo dragon plasma peptide VK25 and its synthetic analogue DRGN-1 | Bacterial load in a carrion-feeding predator | Ranking, on evidence | Activity is shown *in vitro* and in a topical mouse wound model, with no enteric or systemic data and no clinical study. This is E5 on the scale in Section 1.3. | Excluded on evidence, and subsumed by the assessment in Section 6.1 of why individual antimicrobial peptides do not become broad-spectrum systemic agents. | (228) S11 |
| Earthworm (*Eisenia fetida*) lysenin: a sphingomyelin-specific pore-forming defence protein of the coelomic fluid | Microbial challenge in the body cavity | Ranking, on safety | The target lipid is abundant in human plasma membranes. Lysenin lyses mammalian erythrocytes and cultured cells, so the selectivity that makes it a defence molecule in the worm is absent in a human recipient. | Excluded on safety. No redirect at the molecular level; the general lesson is folded into Section 6.1. | (229) S12 |

**References cited in Supplementary Table S1**

S1. Bricelj VM, Connell L, Konoki K, MacQuarrie SP, Scheuer T, Catterall WA, et al. Sodium Channel Mutation Leading to Saxitoxin Resistance in Clams Increases Risk of PSP. Nature (2005) 434(7034):763-7. doi: 10.1038/nature03415.

S2. Karageorgi M, Groen SC, Sumbul F, Pelaez JN, Verster KI, Aguilar JM, et al. Genome Editing Retraces the Evolution of Toxin Resistance in the Monarch Butterfly. Nature (2019) 574(7778):409-12. doi: 10.1038/s41586-019-1610-8.

S3. Ilic Z, Crawford D, Egner PA, Sell S. Glutathione-S-Transferase A3 Knockout Mice Are Sensitive to Acute Cytotoxic and Genotoxic Effects of Aflatoxin B1. Toxicology and Applied Pharmacology (2010) 242(3):241-6. doi: 10.1016/j.taap.2009.10.008.

S4. Eaton DL, Schaupp CM. Of Mice, Rats, and Men: Could Nrf2 Activation Protect against Aflatoxin Heptocarcinogenesis in Humans? Cancer Prevention Research (2014) 7(7):653-7. doi: 10.1158/1940-6207.CAPR-14-0119.

S5. Loh ZH, Ouwerkerk D, Klieve AV, Hungerford NL, Fletcher MT. Toxin Degradation by Rumen Microorganisms: A Review. Toxins (2020) 12(10):664. doi: 10.3390/toxins12100664.

S6. Khan U, Mubariz M, Rezq H, Mahmoud A, Nasir MM, Ul Ain N, et al. Efficacy and Safety of Oxalobacter Formigenes in Patients with Primary Hyperoxaluria: A Systematic Review and Meta-Analysis of Randomized Controlled Trials. Indian Journal of Urology (2025) 41(1):11-19. doi: 10.4103/iju.iju_359_24.

S7. Fischer H, Tschachler E, Eckhart L. Pangolins Lack IFIH1/MDA5, a Cytoplasmic RNA Sensor That Initiates Innate Immune Defense Upon Coronavirus Infection. Frontiers in Immunology (2020) 11:939. doi: 10.3389/fimmu.2020.00939.

S8. Hashimoto T, Horikawa DD, Saito Y, Kuwahara H, Kozuka-Hata H, Shin-I T, et al. Extremotolerant Tardigrade Genome and Improved Radiotolerance of Human Cultured Cells by Tardigrade-Unique Protein. Nature Communications (2016) 7:12808. doi: 10.1038/ncomms12808.

S9. Drabeck DH, Dean AM, Jansa SA. Why the Honey Badger Don't Care: Convergent Evolution of Venom-Targeted Nicotinic Acetylcholine Receptors in Mammals That Survive Venomous Snake Bites. Toxicon (2015) 99:68-72. doi: 10.1016/j.toxicon.2015.03.007.

S10. Rowe AH, Xiao Y, Rowe MP, Cummins TR, Zakon HH. Voltage-Gated Sodium Channel in Grasshopper Mice Defends against Bark Scorpion Toxin. Science (2013) 342(6157):441-6. doi: 10.1126/science.1236451.

S11. Chung EMC, Dean SN, Propst CN, Bishop BM, van Hoek ML. Komodo Dragon-Inspired Synthetic Peptide DRGN-1 Promotes Wound-Healing of a Mixed-Biofilm Infected Wound. npj Biofilms and Microbiomes (2017) 3:9. doi: 10.1038/s41522-017-0017-2.

S12. Sukumwang N, Umezawa K. Earthworm-Derived Pore-Forming Toxin Lysenin and Screening of Its Inhibitors. Toxins (2013) 5(8):1392-401. doi: 10.3390/toxins5081392.

**Supplementary Table S2.** What each level of the two scales asserts, and what it does not. The E scale records translational maturity, which is how far a mechanism has moved toward human use. The M scale records the strength of causal inference for claims about gut bacteria, in the system where the work was done. Neither scale grades the certainty of a result, the size of an effect, or whether an intervention works, and neither is an appraisal of study quality. Those judgements belong to instruments built for them, such as GRADE and the Oxford levels, which are cited in Section 1.3 and are not applied here. The two scales are independent: a claim may stand high on one and low on the other.

| **Level** | **What the label asserts** | **What the label does not assert** | **Nearest established framework** |
| --- | --- | --- | --- |
| E1 | An agent acting on the human node is approved or already in routine use for this or a closely related purpose. | That the agent has been shown to work against foodborne disease. Approval for one indication is not evidence for another, and where the two differ Section 1.3 requires both to be reported. | Phases T3 and T4 of the translational continuum (Section 1.3). |
| E2 | The proposal has been tested in controlled human trials, but is not approved for this use. | That the trial was well conducted, that the effect was large, or that it would replicate. Quality and certainty are not graded on this scale. | Phases T1 and T2. Certainty of the result is the province of GRADE. |
| E3 | Studied in humans, but not in a trial: observational, case-series or mechanistic human work. | That the exposure caused the outcome. The absence of a trial is precisely what the level records. | The boundary of T0 and T1. Study design is ranked by the Oxford levels. |
| E4 | Demonstrated in animals, organoids or germ-free systems, with no human data. | That the finding transfers to humans, and nothing at all about effect size in people. | Phase T0. |
| E5 | Reported only in the source animal, with nothing tested in humans or in any human model. | That the animal work is weak or unreliable. E5 records the absence of human testing, not the quality of what was done in the animal. | Below the translational continuum; added in this review because much comparative work sits here. |
| M1 | The bacteria are found alongside the effect. | Any causal relation whatever. | Association only; the limits of association in microbiome data are set out in Section 1.3. |
| M2 | Removing the microbes changes the effect. | That the microbes are sufficient, or that any single member is responsible. | Depletion evidence. |
| M3 | Transferring the microbes into a germ-free animal reproduces the effect. | That the microbes cause the effect in people. Sufficiency in a gnotobiotic animal is not causation in a human, which is why M3 is a threshold for careful wording rather than proof. | Commensal Koch's postulates (Section 1.3). |
| M4 | One identified microbe, gene or molecule is responsible, shown by removing it or by giving it alone. | That the mechanism operates at the level of the community. Where it does, as in colonisation resistance, the ecological form of the argument applies instead. | Commensal Koch's postulates (Section 1.3). |
| M5 | A controlled human trial supports the causal claim. | That the effect is large or that the result is certain. Magnitude and confidence are not graded here. | Would be appraised by GRADE. |

Supplementary Table S3. The two scales used in this review placed beside established evidence frameworks. The columns do not share a common axis; they are set side by side to make the differences explicit. The E and M scales are independent of one another, so a claim can stand high on one and low on the other. Neither rates efficacy, and neither rates the certainty a body of evidence deserves; those judgements belong to GRADE and to the Oxford levels, which are cited in Section 1.3 and are not applied here. Supplementary Table S2 gives the same distinction level by level.

| **Aspect** | **E1-E5 (this review)** | **M1-M5 (this review)** | **GRADE** | **Oxford Levels** | **T0-T4 continuum** |
| --- | --- | --- | --- | --- | --- |
| Question answered | How far has the mechanism moved toward use in people? | How firmly has cause been established for a claim about gut bacteria? | How much confidence does a body of evidence deserve for one outcome? | How well does a study design answer a clinical question? | What phase of translation has a candidate reached? |
| Unit rated | A mechanism or a proposal | A causal claim about gut bacteria | A body of evidence for a specified outcome | A single study design | A candidate under development |
| Range | E1, approved human use, to E5, observation in the source species alone | M1, association, to M5, controlled human trial | Four levels of certainty, with strength of recommendation reported separately | Five levels, ordered by design within a question type | T0, discovery, to T4, population health impact |
| Rates efficacy? | No | No | Yes, for the specified outcome | Only indirectly, through design | No |
| Rates certainty of evidence? | No | Only in the narrow sense of how firmly cause is established in the system studied | Yes, this is its purpose | Yes, by design rank only | No |
| Applies outside microbiome claims? | Yes | No | Yes | Yes | Yes |
| Anchor point | Approval, or testing in humans | Reconstitution in a germ-free animal (M3 to M4), or a human trial (M5) | Randomised evidence, adjusted up or down | Randomised trial at the top | Clinical use at T3 and T4 |
| Main limit of interpretation | A high grade is not strong evidence, and a low grade is not weak evidence | M3 is sufficiency in a model, not causation in people; single-microbe postulates do not fit community-level effects | Needs a focused question and a named outcome; not built to compare mechanisms across fields | Ranks design, not biological plausibility or readiness for translation | Assumes a candidate already exists; E5 sits below T0 |
